# γδ T cell IFNγ production is directly subverted by *Yersinia pseudotuberculosis* outer protein YopJ in mice and humans

**DOI:** 10.1371/journal.ppat.1010103

**Published:** 2021-12-06

**Authors:** Timothy H. Chu, Camille Khairallah, Jason Shieh, Rhea Cho, Zhijuan Qiu, Yue Zhang, Onur Eskiocak, David G. Thanassi, Mark H. Kaplan, Semir Beyaz, Vincent W. Yang, James B. Bliska, Brian S. Sheridan

**Affiliations:** 1 Department of Microbiology and Immunology, Renaissance School of Medicine, Stony Brook University, Stony Brook, New York, United States of America; 2 Center for Infectious Diseases, Renaissance School of Medicine, Stony Brook University, Stony Brook, New York, United States of America; 3 Department of Medicine, Renaissance School of Medicine, Stony Brook University, Stony Brook, New York, United States of America; 4 Cold Spring Harbor Laboratory, Cold Spring Harbor, New York, United States of America; 5 Department of Microbiology and Immunology, School of Medicine, Indiana University, Indianapolis, Indiana, United States of America; 6 Department of Microbiology and Immunology, Geisel School of Medicine, Dartmouth College, Dartmouth, New Hampshire, United States of America; Stanford University School of Medicine, UNITED STATES

## Abstract

*Yersinia pseudotuberculosis* is a foodborne pathogen that subverts immune function by translocation of *Yersinia* outer protein (Yop) effectors into host cells. As adaptive γδ T cells protect the intestinal mucosa from pathogen invasion, we assessed whether *Y*. *pseudotuberculosis* subverts these cells in mice and humans. Tracking Yop translocation revealed that the preferential delivery of Yop effectors directly into murine Vγ4 and human Vδ2^+^ T cells inhibited anti-microbial IFNγ production. Subversion was mediated by the adhesin YadA, injectisome component YopB, and translocated YopJ effector. A broad anti-pathogen gene signature and STAT4 phosphorylation levels were inhibited by translocated YopJ. Thus, *Y*. *pseudotuberculosis* attachment and translocation of YopJ directly into adaptive γδ T cells is a major mechanism of immune subversion in mice and humans. This study uncovered a conserved *Y*. *pseudotuberculosis* pathway that subverts adaptive γδ T cell function to promote pathogenicity.

## Author summary

Unconventional γδ T cells are a dynamic immune population important for mucosal protection of the intestine against invading pathogens. We determined that the foodborne pathogen *Y*. *pseudotuberculosis* preferentially targets an adaptive subset of these cells to subvert immune function. We found that direct injection of Yersinia outer proteins (Yop) into adaptive γδ T cells inhibited their anti-pathogen functions. We screened all Yop effectors and identified YopJ as the sole effector to inhibit adaptive γδ T cell production of IFNγ. We determined that adaptive γδ T cell subversion occurred by limiting activation of the transcription factor STAT4. When we infected mice with *Y*. *pseudotuberculosis* expressing an inactive YopJ, this enhanced the adaptive γδ T cell response and led to greater cytokine production from this subset of cells to aid mouse recovery. This mechanism of immune evasion appears conserved in humans as direct injection of *Y*. *pseudotuberculosis* YopJ into human γδ T cells inhibited cytokine production. This suggested to us that *Y*. *pseudotuberculosis* actively inhibits the adaptive γδ T cell response through YopJ as a mechanism to evade immune surveillance at the site of pathogen invasion.

## Introduction

Pathogens in the genus *Yersinia* include three species (*Y*. *pestis*, *Y*. *pseudotuberculosis*, and *Y*. *enterocolitica*) that can cause human disease. *Y*. *pseudotuberculosis* and *Y*. *enterocolitica* cause enteric infections [1,2] while *Y*. *pestis* is the causative agent of bubonic, septicemic, and pneumonic plague that has claimed over 200 million human lives [3,4]. Bubonic and septicemic plague is transmitted by blood sucking fleas while aerosols spread the pneumonic plague. Despite vaccine availability [5], and sensitivity to antibiotic treatment, pneumonic plague commonly results in fatality in part due to the rapid course of the infection [6].

Pathogenic *Yersinia spp*. harbor a virulence plasmid that encodes numerous virulence factors to subvert host immune responses, including IFNγ production [7–9]. Immune cell subversion requires *Yersinia* adherence to host cells through bacterial adhesins and translocation of Yersinia outer proteins (Yop) effectors into the host cell cytoplasm by a type III secretion system (T3SS). *Yersinia spp*. predominately target host phagocytes like macrophages, dendritic cells (DC), neutrophils, and B cells to subvert immune function during infection, but injection into other immune populations like conventional T cells has been reported, albeit to a lesser degree than their phagocytic counterparts [10–12]. *Yersinia* virulence factors include components of the T3SS (e.g., YopB) and translocated effectors (e.g., YopJ and YopH). YopB forms a pore in the host cell membrane necessary for translocation of Yop effectors [13,14]. Numerous Yop effectors translocate into host cells to inhibit immune responses and promote *Yersinia spp*. pathogenesis. One notable example is YopJ, an acetyl transferase and a possible cysteine protease that inhibits the mitogen-activated protein kinase (MAPK) pathway and tumor necrosis factor receptor-associated factor (TRAF) ubiquitination [15–19]. YopJ is the major Yop effector responsible for the induction of pyroptosis in macrophages during infection [20] and limits toll-like receptor 4 (TLR4) dependent signaling pathways [21]. While YopJ has no known direct effects on conventional T cell activation, YopP (a YopJ homolog in *Y*. *enterocolitica*) indirectly inhibits T cell priming via DC subversion [22]. YopH has been reported to have direct effects on conventional T cells *in vitro*. Transfection of a YopH expression plasmid into Jurkat or human T cells inhibited T cell receptor (TCR) signaling and promoted T cell apoptosis [23,24]. Additionally, stimulation of Jurkat cells with a YopH deficient *Y*. *pseudotuberculosis* restored T cell signaling and IL-2 production [25,26]. Even in this context, it is notable that many of the downstream targets in the αβ TCR signaling pathway were inhibited at an excessively high (>50) multiplicity of infection (MOI) and *in vivo* relevance is unclear [23,26]. Thus, the role of direct subversion of T cell function, especially among unconventional T cells, by *Yersinia spp*. remains largely unexplored.

γδ T cells make up a large proportion of lymphocytes at barrier surfaces and mucosal tissues including the intestines of mice and humans [27,28]. This is particularly pertinent to infections caused by *Y*. *pseudotuberculosis*, which has evolved to invade the intestinal barrier. The activity of γδ T cells can be modulated by numerous cell-intrinsic and environmental factors like the γδ TCR, cytokines, and co-stimulatory or inhibitory receptors [29]. For example, IL-12 and IL-18 may promote IFNγ production from some γδ T cell subsets whereas IL-1β and IL-23 predominantly drive IL-17A production from other γδ T cell subsets [30–35]. Vγ4Vδ1 (Garman nomenclature [36]) T cells have traditionally been considered an innate-like cell. However, our group recently characterized a long-lived CD27^-^ CD44^hi^ Vγ4Vδ1 T cell memory population in the context of foodborne *Listeria monocytogenes* infection [37,38]. While Vγ4 T cells are typically programmed for IL-17A production, this subset has the multifunctional capacity to produce both IL-17A and IFNγ [37]. Similar observations of IFNγ production were made in clonally expanded Vγ4 T cells in response to *Staphylococcus aureus* in the skin [39]. IFNγ activates macrophages to kill intracellular pathogens or phagocytosed bacteria and induces chemokines that attract immune cells to the site of infection. IFNγ is a critical cytokine in protection from *Y*. *enterocolitica* infection [2,40], *Y*. *pestis* intranasal challenge [41], and associated with protection from *Y*. *pseudotuberculosis* [42]. Interestingly, IFNγ but not IL-17A production from type-3 innate lymphoid cells is critical for the control of foodborne *Y*. *enterocolitica* infection [43]. As such, unconventional T cells like Vγ4 T cells that are ideally placed to provide protection against pathogen invasion at mucosal sites may be particularly relevant to *Yersinia* infections that invade mucosal barriers of the lungs (pneumonic *Y*. *pestis*) and gut (*Y*. *pseudotuberculosis* and *Y*. *enterocolitica*).

Despite a foundational understanding of *Yersinia* pathogenesis, physiologically robust evidence linking *Yersinia* pathogenesis to direct subversion of T cell function is lacking. Here, we uncovered a novel YopJ-dependent immunomodulatory pathway used by *Y*. *pseudotuberculosis* to directly subvert a murine Vγ4Vδ1 anti-microbial response to aid *Y*. *pseudotuberculosis* pathogenesis. *Y*. *pseudotuberculosis* also directly subverted a human Vδ2^+^ T cell IFNγ response, suggesting that this pathway may function similarly in human infection to aid *Y*. *pseudotuberculosis* pathogenesis.

## Results

### Viable *Y*. *pseudotuberculosis* inhibits IFNγ production by adaptive γδ T cells in a YopB- and YadA-dependent manner

**Fig 1:**
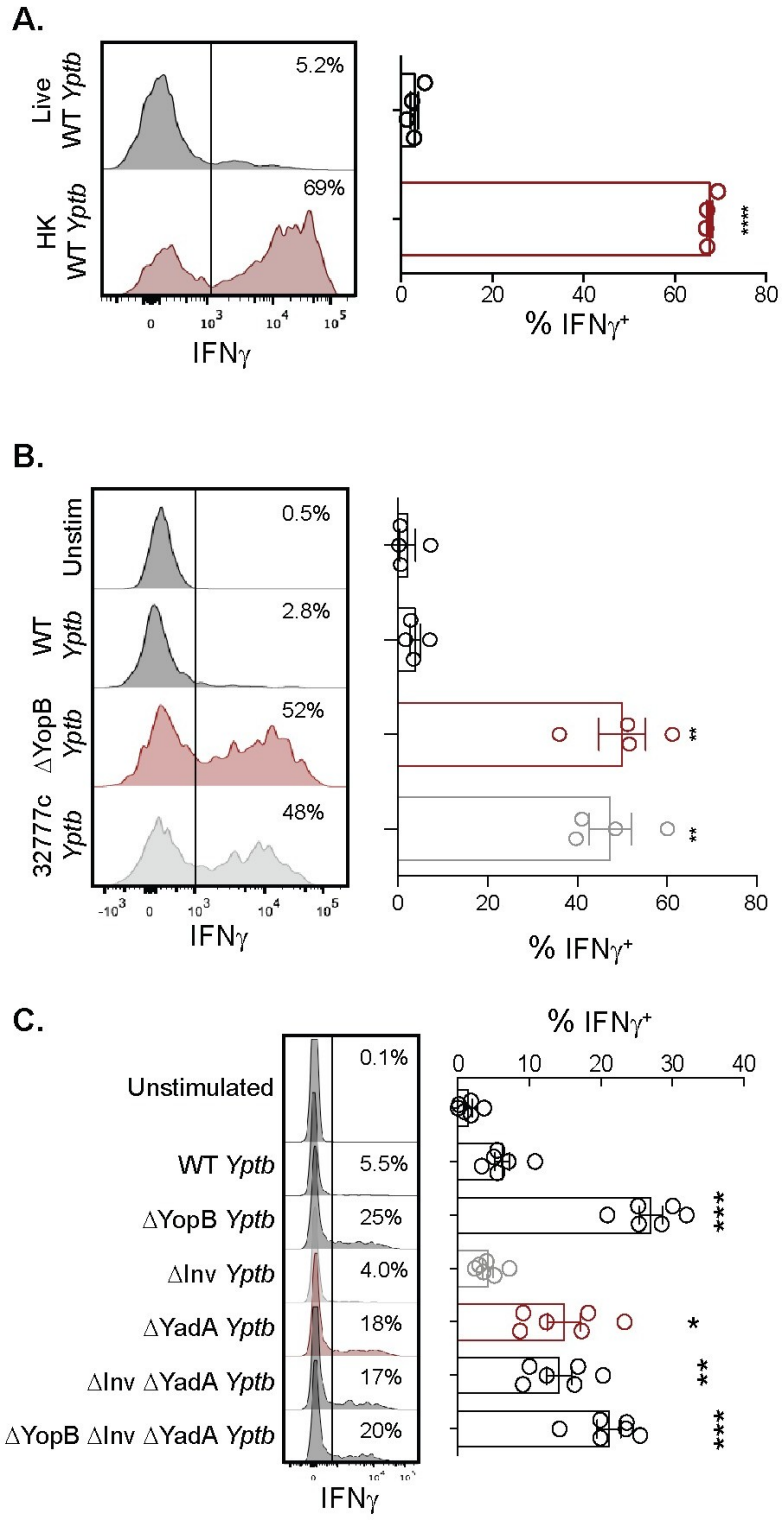
*Y*. *pseudotuberculosis* inhibition of Vγ1.1/2^-^ CD44^hi^ CD27^-^ γδ T cell function is YopB- and YadA-dependent. MLN cell suspensions from *L*. *monocytogenes* infected Balb/c mice were left unstimulated or stimulated with 10 MOI of the indicated *Y*. *pseudotuberculosis* for 24 hours. Antibiotics were given 2 hours post-stimulation and brefeldin A was added for the last 5 hours of stimulation. Vγ1.1/2^-^ CD44^hi^ CD27^-^ γδ T cells were analyzed for IFNγ production. Representative histograms are displayed. (**A**) Cells were stimulated with live or heat-killed (HK) wild-type (WT) *Y*. *pseudotuberculosis*. The graph depicts the mean ± SEM and represents at least two independent experiments with 4 mice/group/experiment. (**B**) Cells were stimulated with live WT, ΔYopB, or 32777c *Y*. *pseudotuberculosis*. The graph depicts the mean ± SEM and represents at least two independent experiments with 4 mice/group/experiment. (**C**) Cells were stimulated with WT, ΔYopB, ΔYadA, ΔInv, ΔInv ΔYadA, or ΔYopB ΔInv ΔYadA *Y*. *pseudotuberculosis*. The graph depicts the mean ± SEM pooled from two independent experiments with 3 mice/group/experiment. ***p < 0.0001, **p < 0.01, and *p < 0.05. An unpaired t-test was used for (**A**) and a repeated measures one-way ANOVA was used for (**B**) and (**C**). Experimental groups were compared to live WT *Y*. *pseudotuberculosis* in (**A**) and WT *Y*. *pseudotuberculosis* in (**B**) and (**C**).

Initial experiments were carried out to determine if *Y*. *pseudotuberculosis* inhibits adaptive γδ T cell function *ex vivo*. To overcome the extremely low number of Vγ4 T cells in gut-associated lymphoid tissues of naïve specific pathogen free (SPF) mice, a previously established *in vivo* methodology was utilized to generate a sizable population of adaptive Vγ4 T cells for *in vitro* manipulation. As such, naïve Balb/c mice were exposed to foodborne *L*. *monocytogenes* and MLN enriched in adaptive γδ T cells were isolated 9 days after infection [37], several days after mice typically clear *L*. *monocytogenes* [44]. MLN single cell suspensions were infected directly *ex vivo* with heat-killed or live wild-type (WT) *Y*. *pseudotuberculosis* (*Yptb*) 32777 (Table 1) at a multiplicity of infection (MOI) of 10 for 2 hours. Antibiotics were then added to prevent overgrowth of the live bacteria, and the cultures were incubated an additional 22 hours. Flow cytometry in conjunction with intracellular cytokine staining was used to assess IFNγ production from Vγ1.1/2^-^ CD44^hi^ CD27^-^ γδ T cells (identifying the adaptive Vγ4 T cell subset [37,38]). Heat-killed *Y*. *pseudotuberculosis* elicited a significantly higher IFNγ response from adaptive Vγ4 T cells than was detectable after stimulation with live *Y*. *pseudotuberculosis* (Fig 1A). This observation suggests that live *Y*. *pseudotuberculosis* subverts adaptive Vγ4 T cell function. The virulence activity of *Y*. *pseudotuberculosis* relies substantially on its T3SS and translocation of Yop effectors into host cells. To determine if the T3SS is required for live *Y*. *pseudotuberculosis* inhibition of γδ T cell function, MLN single cell suspensions were infected with WT *Y*. *pseudotuberculosis* or *Y*. *pseudotuberculosis* that were unable to translocate Yop effectors (ΔYopB) or lacked the virulence plasmid that encodes the T3SS (32777c) (Table 1) [45–47]. γδ T cell function was assessed 24 hours later as described above. Stimulation with live *Y*. *pseudotuberculosis* strains ΔYopB or 32777c restored the IFNγ response of Vγ1.1/2^-^ CD44^hi^ CD27^-^ γδ T cells (Fig 1B), similar to levels seen after stimulation with heat-killed WT *Y*. *pseudotuberculosis* (Fig 1A). These data indicate that *Y*. *pseudotuberculosis* inhibits IFNγ production by Vγ4 T cells in a manner that requires the T3SS and translocation of Yop effectors.

**Table 1:** *Y*. *pseudotuberculosis* strains and mutants used in this study.

*Y*. *pseudotuberculosis*	Notation	Relevant Characteristics	References
32777	WT	*Yptb* wild-type serogroup O:1 strain	[47]
32777c	WT2777c	Virulence pYV-cured derivative of 32777 that lacks the T3SS	[47]
32777 YopJ^C172A^	YopJ^C172A^	Catalytically inactive YopJ	[45]
32777 YopH^R409A^	YopH^R409A^	Catalytically inactive YopH	[45,48,49]
32777 ΔYopB	ΔYopB	Deletion of YopB	[45,46]
32777 YopE^R144A^	YopE^R144A^	Catalytically inactive YopE	[45]
32777 YopT^C139A^	YopT^C139A^	Catalytically inactive YopT	[45]
32777 ΔYopM	ΔYopM	Deletion of YopM	[50]
32777 ΔYpkA	ΔYpkA	Deletion of YpkA	[51]
32777 ΔYopK	ΔYopK	Frameshift mutation in YopK	[52]
32777 YopE/β-lac	WT *Yptb*-βla	YopE TME-1 β-lactamase fusion protein	[53–55]
32777 ΔYopB YopE/β-lac	ΔYopB *Yptb*-βla	Deletion of YopB in the YopE/β-lac	[53–55]
IP2666	WT	*Yptb* wild-type serogroup O:3 strain	[47]
IP40	ΔYopB	IP2666 yopB40 (a stop codon at codon 8 of YopB followed by a frameshift)	[56]
IP2666 ΔInv	ΔInv	Deletion of adhesin and invasin	[12,57]
IP2666 ΔYadA	ΔYadA	Deletion of adhesin and YadA	[12,57]
IP2666 ΔInv ΔYadA	ΔInv ΔYadA	Deletion of adhesin, invasion, and YadA	[12,57]
IP40 ΔInv ΔYadA	ΔYopB ΔInv ΔYadA	Deletion of invasion and YadA in IP40 and pMMB207 mCherry	[12,57]

*Y*. *pseudotuberculosis* adheres to host cells with the bacterial adhesins invasin (Inv) and YadA to translocate effectors through the T3SS [58–60]. For *Y*. *enterocolitica*, both Inv and YadA bind β_1_-integrin either directly or indirectly through the extracellular matrix, respectively [61]. Additionally, β_1_-integrin expressed on host cells is a known adhesion target for *Y*. *pseudotuberculosis* [60,62]. To evaluate the role of these adhesins in the inhibition of γδ T cell function, live *Y*. *pseudotuberculosis* with a deletion of Inv (ΔInv), YadA (ΔYadA), or both (ΔInv ΔYadA) (Table 1) were utilized to infect MLN cell suspensions. Vγ1.1/2^-^ CD44^hi^ CD27^-^ γδ T cells stimulated with ΔInv bacteria produced only minimal IFNγ, comparable to unstimulated cells or cells stimulated with WT (Fig 1C). In contrast, ΔYadA or ΔInv ΔYadA stimulation led to partial restoration of IFNγ production, and stimulation with ΔYopB or ΔYopB ΔInv ΔYadA bacteria led to full restoration of IFNγ production (Fig 1C). Thus, YadA but not Inv contributes to translocation dependent inhibition of IFNγ production by Vγ4 T cells.

### Translocation of Yop effectors into adaptive γδ T cells by *Y*. *pseudotuberculosis* is associated with IFNγ inhibition

**Fig 2:**
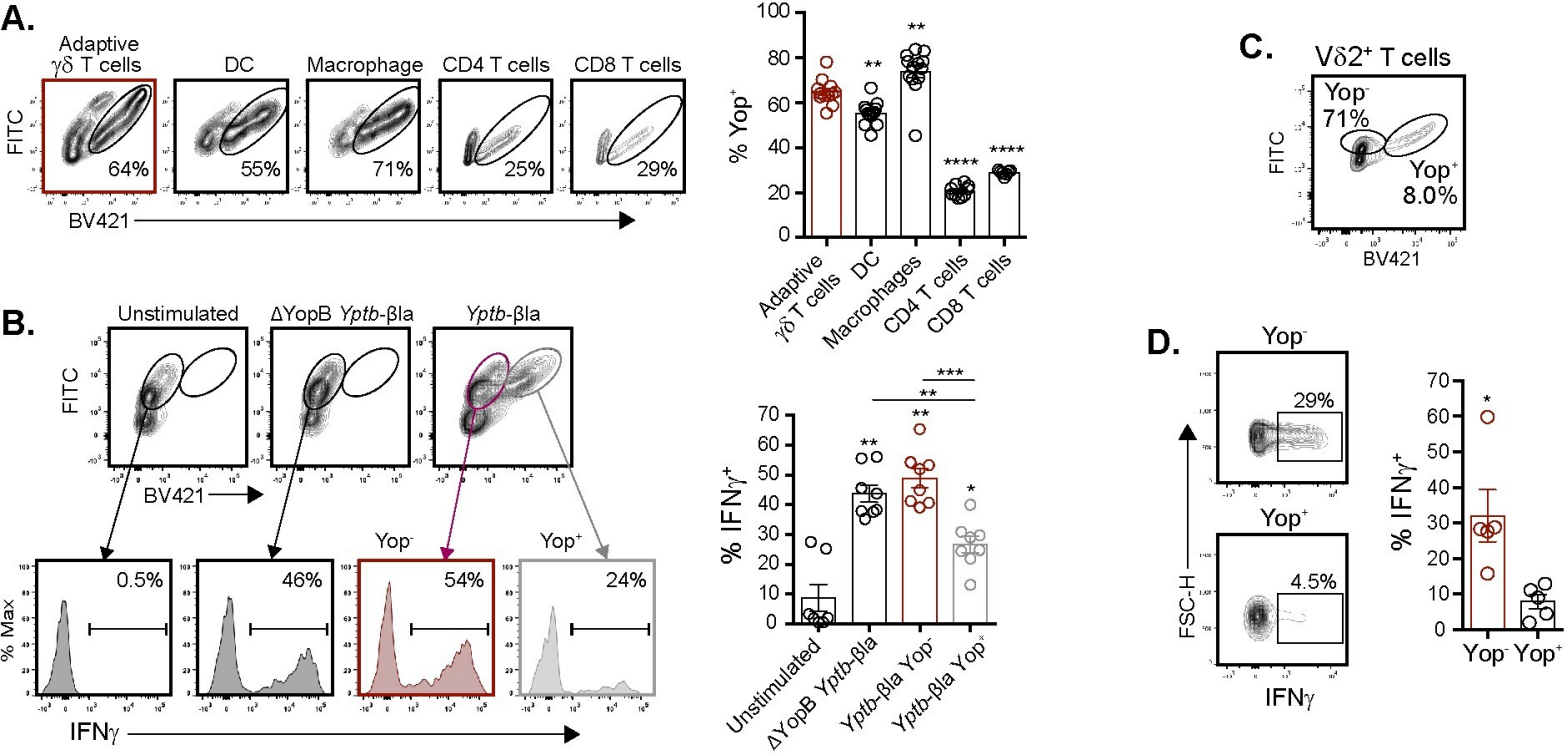
Direct translocation of Yop effectors inhibits the function of murine Vγ4 and human Vδ2^+^ T cells. MLN suspensions from *L*. *monocytogenes* infected mice (**A** and **B**) or human PBMC (**C** and **D**) were left unstimulated or stimulated with WT or ΔYopB *Yptb*-βla as indicated. Cells were loaded with CCF4-AM dye prior to stimulation to measure β-lactamase activity. FITC indicates CCF4-AM loaded cells without translocation (Yop^-^) and BV421 indicates CCF4-AM loaded cells with Yop translocation (Yop^+^). (**A**) Adaptive γδ T cells (Vγ1.1/2^-^ CD44^hi^ CD27^-^ γδ T cells), DC (CD11c^hi^ MHCII^hi^), Macrophages (F4/80^+^ CD11b^+^), and CD4 and CD8 T cells were analyzed for Yop translocation 2 hours post stimulation at an MOI of 10. Representative contour plots are displayed. Yop translocation among the indicated populations is depicted as mean ± SEM and is pooled from 2 experiments with a total of 4–8 mice per group. (**B**) Antibiotics were given 2 hours post-stimulation and brefeldin A was added for the last 5–6 hours of stimulation. Vγ1.1/2^-^ CD44^hi^ CD27^-^ γδ T cells were analyzed for Yop translocation and IFNγ production 24 hours after stimulation. Representative contour plots and histograms are shown. IFNγ production among the indicated populations is depicted as mean ± SEM and is pooled from 3 experiments with a total of 8 mice per group. (**C** and **D**) Antibiotics were given 2 hours post-stimulation and brefeldin A was added for the last 5–6 hours of stimulation. Vδ2^+^ T cells were analyzed for Yop translocation and IFNγ production post stimulation. Representative contour plots are displayed and IFNγ production is quantified among Yop^+^ or Yop^-^ Vδ2^+^ T cells. The graph depicts mean ± SEM and is pooled from 3 experiments with 5 healthy donors per group. ****p < 0.0001, ***p < 0.001, **p < 0.01, and *p < 0.05. An ordinary one-way ANOVA was used for (**A**), a repeated measures one-way ANOVA was used for (**B**), and a paired t-test was used for (**D**). Comparisons were performed to adaptive γδ T cells in (**A**), to unstimulated or as depicted in figure in (**B**), and to Yop^+^ in (**C**).

To determine if *Y*. *pseudotuberculosis* can translocate Yop effectors into adaptive Vγ4 T cells, a WT strain expressing a YopE-β-lactamase fusion protein (*Yptb*-βla) in conjunction with a FRET-based β-lactamase reporter assay was used [63]. YopE translocation into target cells can be readily assessed by a change in fluorescence using flow cytometry. Thus, translocation of the YopE-β-lactamase fusion protein reports Yop effector translocation by emission in the blue range (Yop^+^) or lack thereof by emission in the green range (Yop^-^) [10,64,65]. A YopB deficient β-lactamase *Y*. *pseudotuberculosis* reporter (ΔYopB *Yptb-*βla) was used as a translocation deficient control. Stimulation of MLN cell suspensions with WT or ΔYopB *Yptb-*βla confirmed reporter activity at various MOI (S1A and S1B Fig). Two hours post stimulation with WT *Yptb-*βla, the majority of Vγ1.1/2^-^ CD44^hi^ CD27^-^ γδ T cells were positive for Yop effector translocation (Fig 2A). Yop translocation into Vγ1.1/2^-^ CD44^hi^ CD27^-^ γδ T cells was comparable to known DC and macrophage targets (Fig 2A). Additionally, Yop translocation was more efficient into Vγ1.1/2^-^ CD44^hi^ CD27^-^ γδ T cells than CD4 or CD8 T cells (Fig 2A). *Y*. *pseudotuberculosis* also preferentially targeted Vγ1.1/2^-^ CD44^hi^ CD27^-^ γδ T cells over CD44^-^ γδ T cells and activated phenotype CD4 or CD8 T cells for Yop translocation (S1C Fig). YadA and Inv promote *Yersinia* adherence by direct or indirect interactions with the β_1_-integrin [66–69]. Analysis of β_1_-integrin expression on Vγ1.1/2^-^ CD44^hi^ CD27^-^ γδ T cells, CD4 T cells, and CD8 T cell revealed that most Vγ1.1/2^-^ CD44^hi^ CD27^-^ γδ T cells expressed the β1-integrin (S2A Fig). In contrast, most conventional CD4 and CD8 T cells did not express the β_1_-integrin. In addition, use of the WT *Yptb-*βla reporter for Yop translocation demonstrated that Yop translocation was associated with higher β_1_-integrin expression among γδ T cells (S2B Fig). Thus, *Y*. *pseudotuberculosis* selectively targets adaptive γδ T cells for Yop translocation among a diverse group of immune populations assessed in an *ex vivo* culture system.

As adaptive Vγ4 T cells were directly targeted with Yop effector translocation, WT *Yptb*-βla was utilized to determine whether Vγ1.1/2^-^ CD44^hi^ CD27^-^ γδ T cells that contained Yop effectors were functionally impaired. An MOI of 1 was used as it provided similarly sized populations of Vγ1.1/2^-^ CD44^hi^ CD27^-^ γδ T cells that did or did not contain translocated effectors from the same culture conditions (S1B Fig). Among WT *Yptb*-βla stimulated cells, Yop^+^ Vγ1.1/2^-^ CD44^hi^ CD27^-^ γδ T cells had reduced IFNγ production as compared to their Yop^-^ counterparts (Fig 2B). To extend these results, the ability of *Y*. *pseudotuberculosis* to translocate Yop effectors into human γδ T cells and inhibit IFNγ production was assessed in peripheral blood mononuclear cells (PBMC) cultures stimulated with the WT *Yptb*-βla reporter. Approximately 8% of human Vδ2^+^ T cells were Yop^+^ and these cells had significantly reduced IFNγ production as compared to the Yop^-^ counterparts (Fig 2C and 2D). These data indicate that *Y*. *pseudotuberculosis* is capable of translocating Yop effectors into γδ T cell subsets and inhibiting IFNγ production in mice and humans.

### YopJ is necessary for *Y*. *pseudotuberculosis* to inhibit IFNγ production in adaptive γδ T cells

**Fig 3:**
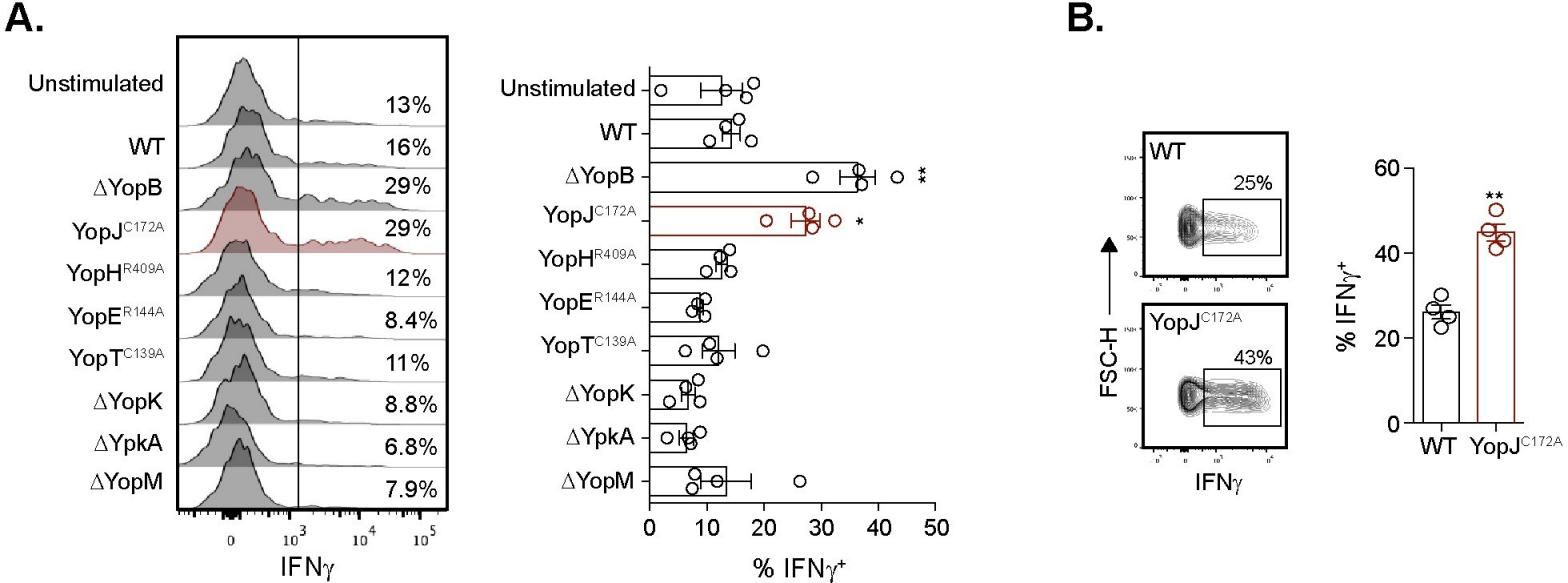
YopJ is necessary for inhibition of IFNγ production in murine Vγ4 and human Vδ2^+^ T cells. (**A**) MLN from *L*. *monocytogenes* infected mice were left unstimulated or stimulated with 10 MOI of the indicated *Y*. *pseudotuberculosis* for 24 hours. Antibiotics were given 2 hours post-stimulation and brefeldin A was added for the last 5–6 hours. Vγ1/2^-^ CD44^hi^ CD27^-^ γδ T cells were analyzed for IFNγ post stimulation. Representative histograms are displayed. The graph depicts mean ± SEM and represents at least two independent experiments with 2–4 mice per group. (**B**) Human PBMC were stimulated with 1 MOI of WT or YopJ^C172A^ *Y*. *pseudotuberculosis* for 24 hours. Antibiotics were given 2 hours post-stimulation. Brefeldin A was added for the last 5–6 hours of stimulation. Vδ2^+^ γδ T cells were analyzed for IFNγ production post stimulation. Representative flow plots gated on Vδ2^+^ T cells are displayed. The graph depicts mean ± SEM and is pooled from 2 experiments with 4 healthy donors. **p < 0.01 and *p < 0.05. A repeated measures one-way ANOVA was used for (**A**) and a paired t-test was used for (**B**). Experimental groups were compared to WT *Y*. *pseudotuberculosis*.

As multiple effectors are translocated into target cells, a panel of *yop* mutant *Y*. *pseudotuberculosis* (Table 1) [45] was screened to determine if individual Yop effectors inhibit IFNγ production. Similar to the ΔYopB mutant, stimulation with a catalytically inactive YopJ (YopJ^C172A^) mutant that lacks acetyl transferase activity, but not other mutant *Y*. *pseudotuberculosis*, restored IFNγ production in Vγ1.1/2^-^ CD44^hi^ CD27^-^ γδ T cells (Fig 3A). The C172A mutation in YopJ prevents YopJ mediated inhibition of MAPK and NF-κB signaling pathways by abolishing its serine and threonine acetylation activity [70]. A similar restoration of IFNγ production was observed in human Vδ2^+^ T cells from PBMC of healthy donors upon YopJ^C172A^ *Y*. *pseudotuberculosis* stimulation (Fig 3B). Thus, the YopJ effector is responsible for inhibition of IFNγ production from murine Vγ4 and human Vδ2^+^ T cells.

### YopJ inhibits expression of multiple genes, including *ifng*, in adaptive γδ T cells

**Fig 4:**
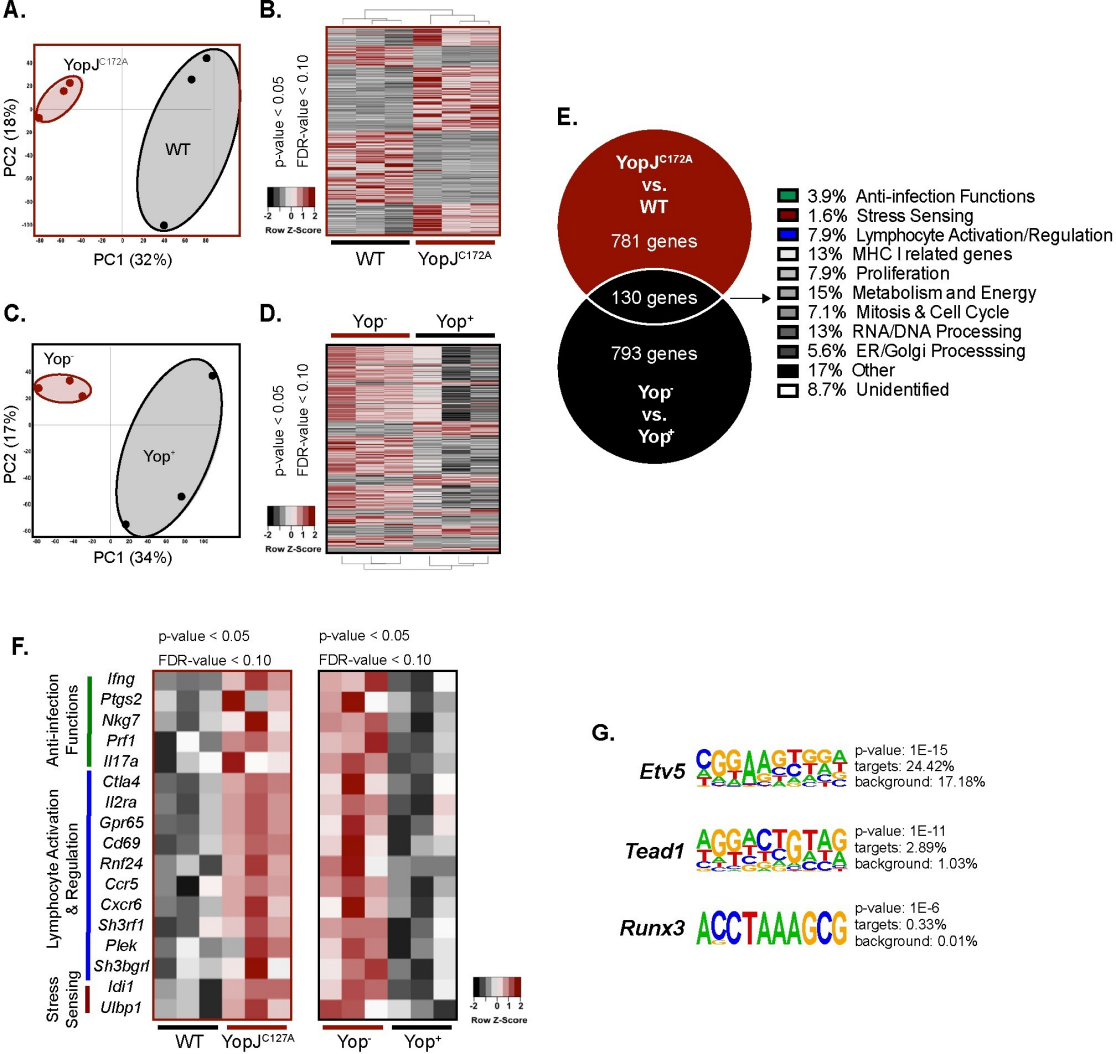
YopJ translocation leads to the inhibition of a broad anti-microbial gene response from Vγ4 T cells. (**A** and **B**) MLN suspensions from *L*. *monocytogenes* infected mice were stimulated with 10 MOI of WT or YopJ^C172A^ *Y*. *pseudotuberculosis* for 24 hours. Antibiotics were given 2 hours post-stimulation. Five hundred Vγ1.1/2^-^ CD44^hi^ CD27^-^ γδ T cells from each stimulation were flow sorted and processed for RNA sequencing. (**A**) PCA plots are depicted for similarity of groups YopJ^C172A^ and WT *Y*. *pseudotuberculosis* stimulated Vγ1.1/2^-^ CD44^hi^ CD27^-^ γδ T cells. (**B**) Heat maps are depicted for differentially expressed genes of YopJ^C172A^ or WT *Y*. *pseudotuberculosis* stimulated Vγ1.1/2^-^ CD44^hi^ CD27^-^ γδ T cells. (**C** and **D**) MLN suspension from *L*. *monocytogenes* infected mice were stimulated with 1 MOI of WT *Yptb*-βla. Five hundred Yop^+^ or Yop^-^ Vγ1.1/2^-^ CD44^hi^ CD27^-^ γδ T cells were flow sorted and processed for RNA sequencing. (**C**) PCA plots are depicted for similarity of Yop^+^ or Yop^-^ Vγ1.1/2^-^ CD44^hi^ CD27^-^ γδ T cells. (**D**) Heat maps are depicted for differentially expressed genes of Yop^-^ or Yop^+^ stimulated Vγ1.1/2^-^ CD44^hi^ CD27^-^ γδ T cells. (**E**-**G**) A Venn diagram of differentially expressed genes (higher) that overlapped between RNA sequencing analyses favoring YopJ^C172A^ *Y*. *pseudotuberculosis* stimulation or Yop^-^ cells is displayed. Shared genes were categorized by gene function. (**F**) The heat map highlights differentially expressed genes among Vγ1.1/2^-^ CD44^hi^ CD27^-^ γδ T cells from the indicated stimulations and categories. (**G**) Homer motif analysis was performed on the RNA sequencing dataset. Motifs and associated genes to YopJ^C172A^ stimulated Vγ1.1/2^-^ CD44^hi^ CD27^-^ γδ T cells are highlighted. Each experiment was performed with 3 biologic samples per group. Cutoffs for significant genes are p < 0.05 and FDR < 0.10.

To uncover mechanisms by which YopJ inhibits IFNγ production, the transcriptome of cell sorter-purified Vγ1.1/2^-^ CD44^hi^ CD27^-^ γδ T cells after WT or YopJ^C172A^ *Y*. *pseudotuberculosis* stimulation of MLN cells was assessed by RNA-Seq. Principal component analysis revealed unique gene expression clustering, and approximately 900 genes were expressed at higher levels in the YopJ^C172A^ stimulation as compared to WT *Y*. *pseudotuberculosis* (Fig 4A and 4B). These differentially expressed genes may include genes that are directly inhibited by YopJ activity in Vγ1.1/2^-^ CD44^hi^ CD27^-^ γδ T cells or indirectly inhibited by YopJ activity in other cells such as DC or macrophages. To resolve this, the WT *Yptb*-βla reporter provided an opportunity to evaluate the molecular changes elicited by the activity of translocated Yop in adaptive γδ T cells. The transcriptome of sort purified Yop^-^ and Yop^+^ Vγ1.1/2^-^ CD44^hi^ CD27^-^ γδ T cells after WT *Yptb*-βla stimulation was assessed by RNA-Seq. Principal component analysis revealed unique gene expression clustering, and approximately 900 genes were more highly expressed in Yop^-^ vs Yop^+^ Vγ1.1/2^-^ CD44^hi^ CD27^-^ γδ T cells after WT *Yptb*-βla stimulation (Fig 4C and 4D). Overlapping gene expression profiles from the two datasets were assessed to narrow the analysis to direct YopJ effects on Vγ1.1/2^-^ CD44^hi^ CD27^-^ γδ T cells. This comparison revealed 130 genes that were differentially expressed in both datasets, suggesting they are regulated directly by translocated YopJ in adaptive Vγ4 T cells (Fig 4E). These genes were categorized into different groups depending on their known functions. Some differentially expressed genes play a particular role in anti-infection functions (3.9%), stress sensing (1.6%), and lymphocyte activation/regulation (7.9%), genes that may be important for protective T cell responses (Fig 4E and 4F). Among these genes, IFNγ was the single most significant differentially expressed gene suggesting it is a major target of direct YopJ-mediated inhibition of adaptive γδ T cell function (Fig 4F). Differentially expressed genes among those that promote antimicrobial function included several that are important in augmenting type-1 and -3 inflammation in T cells. For example, *Ptgs2* (encodes cyclooxygenase-2, COX2), *Nkg7* (natural killer cell granule protein 7), *Prf1* (perforin-1), and *Il17a* (IL-17A) appear to be regulated directly by translocated YopJ in adaptive Vγ4 T cells (Fig 4F) [71–73]. However, analysis of IL-17A protein after stimulation of MLN cell suspensions with YopJ^C172A^, WT, and ΔYopB *Y*. *pseudotuberculosis* demonstrated that YopJ did not regulate IL-17A production from Vγ1.1/2^-^ CD44^hi^ CD27^-^ γδ T cells (S4 Fig). Some of the observed differentially expressed genes are important in the activation status of T cells (e.g., *Il2ra*, *Ctla4*, and *Cd69*) and suggest that translocated YopJ may limit the activation of adaptive Vγ4 T cells. There was also a notable impact (48.0%) on genes associated with cell proliferation, metabolism and energy, mitosis and cell cycle, RNA/DNA processing, and ER/Golgi processing (Figs S3A and S3B and 4E), suggesting that YopJ influences the adaptive γδ T cell transcriptional profile more broadly than just targeting the IFNγ pathway. Genes that were differentially expressed upon WT *Y*. *pseudotuberculosis* stimulation or among Yop^+^ cells also suggest that many of the processes associated with immune responses and cellular activity were regulated by YopJ (S3C–S3E Fig). Collectively, YopJ appears to regulate the expression of many genes associated with T cell function in Vγ1.1/2^-^ CD44^hi^ CD27^-^ γδ T cells, suggesting that adaptive Vγ4 T cells are broadly constrained in their immune functions by *Y*. *pseudotuberculosis*.

### YopJ inhibits the IL-12p40-mediated STAT4 pathway in adaptive γδ T cells

To gain potential mechanistic insights into YopJ inhibition of IFNγ production and other Vγ1.1/2^-^ CD44^hi^ CD27^-^ γδ T cell functions, a motif discovery algorithm designed for regulatory element analysis was utilized to assess our RNA sequencing results [74]. Several transcription factor binding motifs related to IFNγ signaling were differentially expressed after YopJ^C172A^ *Y*. *pseudotuberculosis* but not WT *Y*. *pseudotuberculosis* stimulation including members of the E twenty-six (ETS)-domain family, Krüppel-like factor and specificity protein (KLF/SP) transcription factor gene family, and the interferon regulatory factors (IRF) family of transcription factors (S5A Fig). IRF8 protein was validated after WT, ΔYopB, and YopJ^C172A^ *Y*. *pseudotuberculosis* stimulation. Indeed, a higher percentage of Vγ4 T cells expressed IRF8 protein after stimulation with ΔYopB compared to WT *Y*. *pseudotuberculosis* stimulation (S5B Fig). Stimulation with YopJ^C172A^ *Y*. *pseudotuberculosis* was also able to partially restore IRF8 levels to those seen after ΔYopB *Y*. *pseudotuberculosis* stimulation (S5B Fig). IRF8 was also impacted by IL-12p40 blockade, which signals through signal transducer and activator of transcription 4 (STAT4) (S5B Fig). Interestingly, a number of transcription factor binding motifs downstream of STAT4 signaling were enriched including *Etv5*, *Runx3*, and *Tead1* (Fig 4G) [75–78]. The RNAseq and homer motif analyses were also compared to an existing STAT4 ChIP-on-chip [79]. 7 genes identified from our main analyses (Figs 4F and 4G and S5B) were STAT4 target genes (S5C Fig). In summary, transcriptional profiling revealed a global subversion of anti-pathogen immune functions that may be associated with YopJ subversion of STAT4 activity.

**Fig 5:**
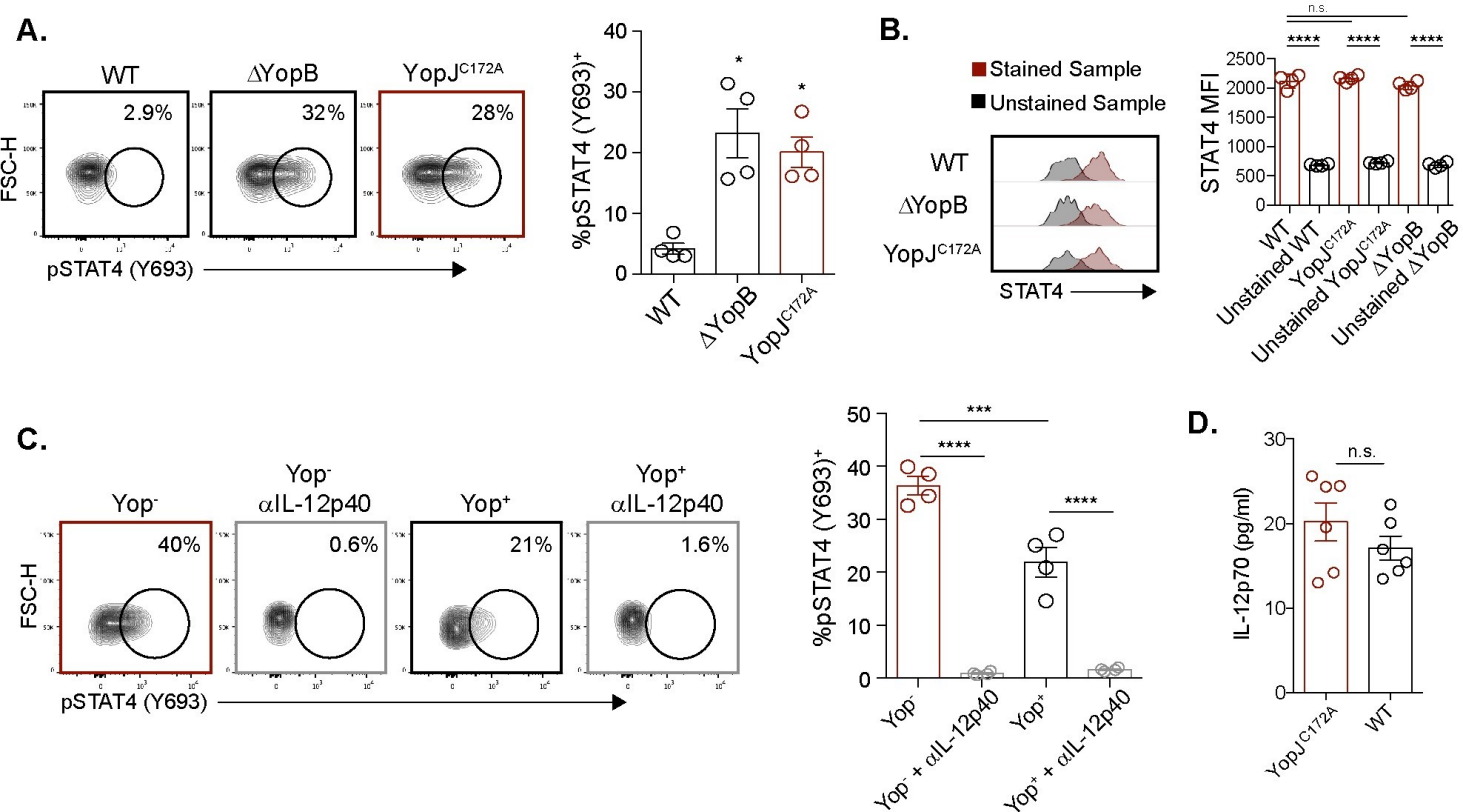
YopJ inhibits IL-12p40 mediated STAT4 phosphorylation. (**A**) MLN cell suspensions from *L*. *monocytogenes* infected mice were stimulated with 10 MOI of WT, YopJ^C172A^, or ΔYopB *Y*. *pseudotuberculosis* for 6 hours. Antibiotics were given 2 hours after stimulation. Vγ1.1/2^-^ CD44^hi^ CD27^-^ γδ T cells were analyzed for pSTAT4 after stimulation. Representative contour plots are displayed. The graph depicts mean ± SEM and represents at least two independent experiments with 2–4 mice per group. (**B**) The same experimental setup was used as in (**A**), but Vγ1.1/2^-^ CD44^hi^ CD27^-^ γδ T cells were analyzed for STAT4 protein after stimulation. Representative plots for mean fluorescent intensity (MFI) are displayed. The graph depicts mean ± SEM and represents two independent experiments with 2 mice per group. (**C**) MLN suspensions from *L*. *monocytogenes* infected mice were either treated or untreated with IL-12/23p40 neutralizing antibody prior to stimulation with 1 MOI of WT *Yptb*-βla for 6 hours. Antibiotics were given 2 hours post-stimulation. Vγ1.1/2^-^ CD44^hi^ CD27^-^ γδ T cells were analyzed for pSTAT4 after stimulation. Representative contour plots are displayed. The graph depicts mean ± SEM and represents at least two independent experiments with 2–4 mice per group. (**D**) MLN cell suspensions from *L*. *monocytogenes* infected mice were stimulated with 10 MOI of WT or YopJ^C172A^ *Y*. *pseudotuberculosis* for 24 hours. Antibiotics were given 2 hours after stimulation. Supernatants were collected 24 hours post stimulation and IL-12p70 concentration was determined via ELISA. ****p < 0.0001, ***p < 0.001, **p < 0.01, and *p < 0.05. A repeated measures one-way ANOVA was used for (**A**-**C**). Comparisons were performed to WT *Y*. *pseudotuberculosis* in (**A**) and as depicted in (**B**-**D**).

IL-12 signaling leads to STAT4 phosphorylation and formation of STAT4-STAT4 homodimers that re-localize to the nucleus where they directly bind to the *Ifng* promoter to induce IFNγ expression [79–81]. To determine whether YopJ inhibits IFNγ production by interfering with the STAT4 pathway, STAT4 protein and phosphorylation were assessed by flow cytometry of MLN cells stimulated with *Y*. *pseudotuberculosis*. STAT4 phosphorylation was analyzed 6 hours after stimulation with WT, ΔYopB, or YopJ^C172A^ *Y*. *pseudotuberculosis*. Consistent with suppression of IFNγ production and the RNA-Seq analysis, WT *Y*. *pseudotuberculosis* significantly reduced the percentage of pSTAT4^+^ CD44^hi^ CD27^-^ γδ T cells as compared to ΔYopB and YopJ^C172A^ *Y*. *pseudotuberculosis* (Fig 5A). However, STAT4 protein levels were the same in all three infection conditions (WT, ΔYopB, and YopJ^C172A^ *Y*. *pseudotuberculosis*) (Fig 5B). Flow cytometry antibodies for STAT4 protein were validated by comparing STAT4 from WT and STAT4 KO splenocytes (S5D Fig). These data suggest that STAT4 phosphorylation but not protein is decreased upon YopJ translocation. STAT4 phosphorylation was also evaluated using the *Yptb*-βla reporter system described above. Among CD44^hi^ CD27^-^ γδ T cells, Yop^-^ cells had a higher percentage of pSTAT4^+^ cells compared to Yop^+^ cells suggesting intrinsic Yop mediated inhibition of STAT4 phosphorylation levels (Fig 5C). Additionally, as STAT4 phosphorylation is downstream of IL-12 signaling, an anti-IL-12/23p40 subunit antibody (anti-p40) was used to determine whether IL-12 signals in the environment regulated STAT4 phosphorylation after *Y*. *pseudotuberculosis* stimulation. Indeed, IL-12/23p40 neutralization abrogated STAT4 phosphorylation levels regardless of Yop translocation (Fig 5C). As IL-12/23p40 was required to elicit IFNγ production from adaptive Vγ4 T cells in the culture conditions, we assessed whether YopJ^C172A^ *Y*. *pseudotuberculosis* stimulation modulated IL-12p70. The concentration of IL-12p70 was comparable between WT and YopJ^C172A^ *Y*. *pseudotuberculosis* stimulated cultures (Fig 5D). Thus, changes in IL-12 were unlikely to contribute to adaptive Vγ4 T cell subversion *in vitro*. To understand the role of YopJ and IL-12 on Vγ1.1/2^-^ CD44^hi^ CD27^-^ γδ T cells in a more simplified system, purified γδ T cells were stimulated with YopJ^C172A^ *Y*. *pseudotuberculosis* in the presence of excessive IL-12p70. Adaptive Vγ4 T cells were unable to produce IFNγ in response to YopJ^C172A^ *Y*. *pseudotuberculosis* and IL-12p70 (S6A Fig). Finally, we assessed whether the addition of IL-12p70 could overcome the YopJ mediated inhibition of IFNγ production after WT *Y*. *pseudotuberculosis* stimulation. While a supraphysiologic level of IL-12p70 (50 ng/ml) was able to partially overcome YopJ mediated inhibition, lower levels of IL-12p70 addition (2 and 10 ng/ml) were unable to overcome YopJ mediated inhibition (S6B Fig). Importantly, these latter concentrations were orders of magnitude higher than those detected in our culture conditions. Thus, IL-12 is not sufficient to induce IFNγ production from adaptive Vγ4 T cells. Collectively, these results suggest that *Y*. *pseudotuberculosis* stimulation elicits IL-12 production to promote adaptive Vγ4 T cell IFNγ responses, and that YopJ translocation into adaptive Vγ4 T cells inhibits IL-12 mediated STAT4 phosphorylation.

### Foodborne infection with YopJ^C172A^ *Y*. *pseudotuberculosis* induces IFNγ production in adaptive Vγ4 T cells

**Fig 6:**
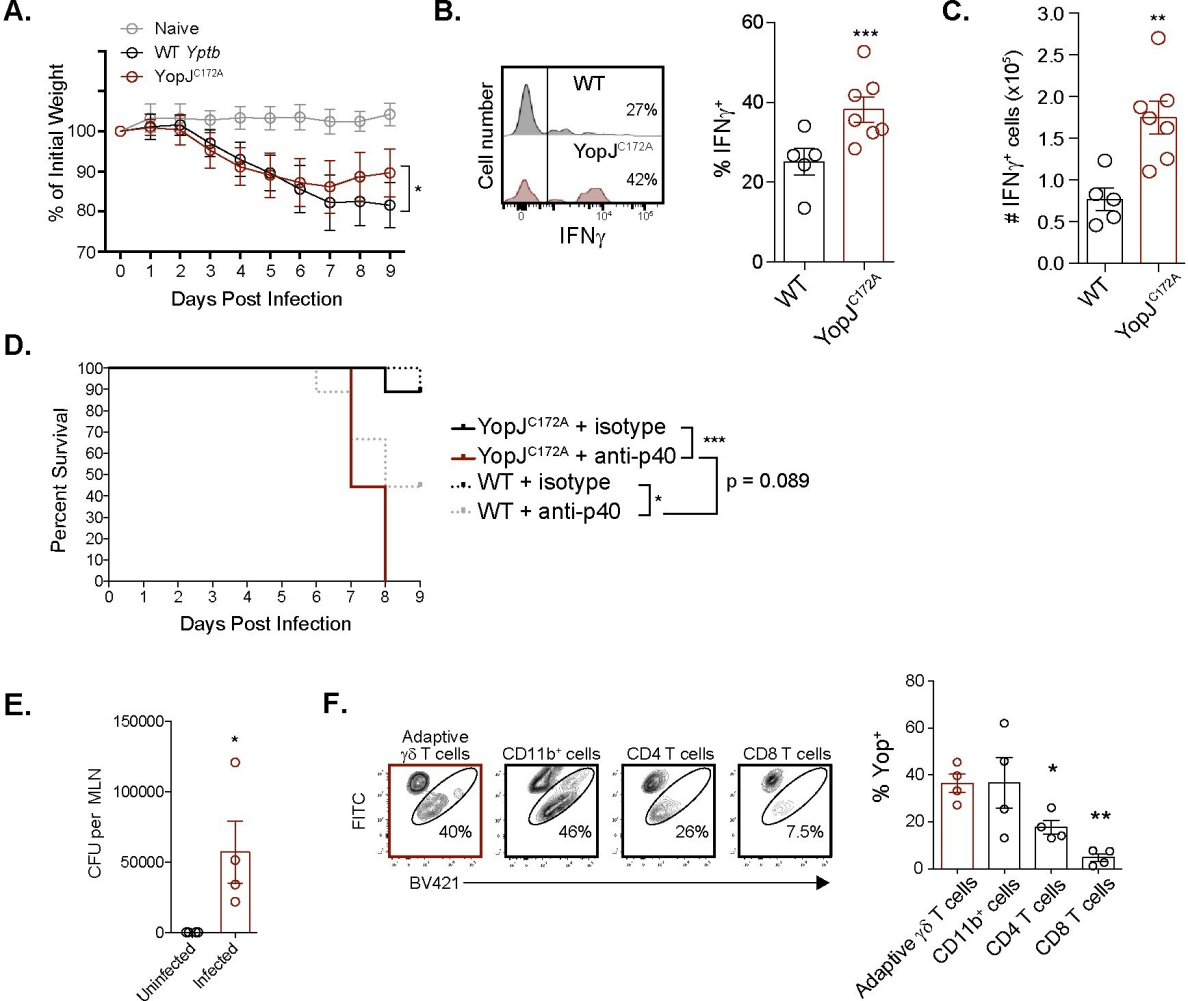
Foodborne YopJ^C172A^ *Y*. *pseudotuberculosis* infection elicits an IFNγ response from Vγ4 T cells. (**A**-**E**) Balb/c mice were foodborne infected with WT (2-4x10^7^ CFU) or YopJ^C172A^ *Y*. *pseudotuberculosis* (2-4x10^8^ CFU). (**A**) Mouse weight was assessed daily after infection. (**B** and **C**) Nine days post infection, MLN were processed into single cell suspensions and stimulated with PMA/ionomycin in the presence of brefeldin A for 4 hours. Vγ1.1/2^-^ CD44^hi^ CD27^-^ γδ T cells were analyzed for IFNγ production. Representative histograms are displayed. Graphs represent mean ± SEM and are pooled from 3 experiments with a total of 5–8 mice per group. (**D**) Mouse survival was assessed daily after infection. anti-IL-12p40 antibody was administered at 0.2 mg/mouse on 0, 2, 4, and 6 days post infection. A Kaplan-Meier survival plot is shown. Study endpoint was 9 days post infection. The data represent 2 independent experiments with a total of 9 mice per group. (**E** and **F**) Balb/c mice were foodborne infected with 2x10^9^ CFU *L*. *monocytogenes* to elicit a Vγ1.1/2^-^ CD44^hi^ CD27^-^ γδ T cell population *in vivo*. 30 days post infection, immunized mice were foodborne infected with WT *Yptb*-βla (2-4x10^9^ CFU) or left uninfected. (**E**) CFU of WT *Yptb*-βla were enumerated in MLN 3 days post WT *Yptb*-βla infection. (**F**) Three days post foodborne WT *Yptb*-βla infection, Vγ1.1/2^-^ CD44^hi^ CD27^-^ γδ T cells from the MLN were analyzed for Yop translocation using the CCF4-AM assay. Representative contour plots are shown. Yop translocation (Yop^+^) among the indicated populations is depicted as mean ± SEM and is pooled from 2 experiments with a total of 4 mice per group. ***p < 0.001, **p < 0.01, and *p < 0.05. A repeated measures one-way ANOVA was used for (**A** and **F**) and an unpaired t-test was used for (**B**, **C**, and **E**). Experimental groups were compared to WT *Y*. *pseudotuberculosis* in (**A**-**C**), uninfected controls in (**E**), and Vγ1.1/2^-^ CD44^hi^ CD27^-^ γδ T cells in (**F**). A logrank test was used for survival curves in (**D**).

To determine whether YopJ subverts adaptive γδ T cell function *in vivo*, foodborne infection with WT and YopJ^C172A^ *Y*. *pseudotuberculosis* was performed on naïve Balb/c mice. As YopJ^C172A^ *Y*. *pseudotuberculosis* is attenuated *in vivo* [82], a one log higher (2-4x10^8^ CFU) infection dose of YopJ^C172A^ *Y*. *pseudotuberculosis* was administered to normalize the internal bacteria burdens in the MLN between infection groups (S7A Fig). While mice infected with WT and YopJ^C172A^ *Y*. *pseudotuberculosis* lost a similar amount of weight, mice infected with YopJ^C172A^ *Y*. *pseudotuberculosis* recovered slightly faster (Fig 6A). MLN were isolated 9 days after infection to evaluate adaptive γδ T cell function. Consistent with the *ex vivo* stimulation of *L*. *monocytogenes*-elicited Vγ4 T cells with *Y*. *pseudotuberculosis*, Vγ1.1/2^-^ CD44^hi^ CD27^-^ γδ T cells from the MLN of YopJ^C172A^ *Y*. *pseudotuberculosis* infected mice displayed enhanced IFNγ production when stimulated *ex vivo* compared to their WT *Y*. *pseudotuberculosis* infected counterparts (Fig 6B and 6C). When the same infectious doses were used for both WT and YopJ^C172A^ *Y*. *pseudotuberculosis* (5x10^7^ CFU), Vγ1.1/2^-^ CD44^hi^ CD27^-^ γδ T cells from the MLN of YopJ^C172A^ *Y*. *pseudotuberculosis* infected mice also displayed enhanced IFNγ production compared to their WT *Y*. *pseudotuberculosis* infected counterparts (S7B Fig). These experiments demonstrate that the increased IFNγ observed was not a result of a higher infectious dose nor of a higher bacteria burden at the time of analysis. As IL-12/23p40 was required for adaptive Vγ4 T cell IFNγ production *in vitro* (Fig 5), the impact of IL-12/23p40 was assessed *in vivo*. Naïve Balb/c mice were infected with WT or YopJ^C172A^ *Y*. *pseudotuberculosis* and treated with an IL-12/23p40 neutralizing antibody or isotype control. All YopJ^C172A^ *Y*. *pseudotuberculosis* infected mice treated with anti-IL-12/23p40 succumbed by day 8 post infection (Fig 6D). This data suggests that IL-12 promotes the protective capacity of catalytically inactive YopJ. Similarly, IL-12 contributed to the protection of mice infected with WT *Y*. *pseudotuberculosis*. Serum was also collected on days 2, 4 and 6 after infection to assess circulating IL-12p70 levels. IL-12p70 was detectable 6 days after YopJ^C172A^ *Y*. *pseudotuberculosis* infection but was mostly below the limit of detection after WT *Y*. *pseudotuberculosis* infection (Fig 6E). Collectively, these data suggest that IL-12 is important in the protection of mice infected with WT or YopJ^C172A^ *Y*. *pseudotuberculosis*.

Foodborne infection with WT *Yptb*-βla was performed to determine whether *Y*. *pseudotuberculosis* could target adaptive Vγ4 T cells with Yop translocation *in vivo*. Balb/c mice were foodborne infected with *L*. *monocytogenes* to elicit a population of Vγ1.1/2^-^ CD44^hi^ CD27^-^ γδ T cells as described previously [37,83]. After a return to homeostasis at 30 days post *L*. *monocytogenes* infection, mice were foodborne infected with WT *Yptb*-βla. *Y*. *pseudotuberculosis* burden was assessed in the MLN 3 days post foodborne infection to determine whether WT *Yptb*-βla could establish a productive infection where Vγ4 T cells reside. Indeed, infected mice contained detectable *Y*. *pseudotuberculosis* in the MLN 3 days after foodborne infection (Fig 6F). Analysis of the translocation of Yop into Vγ1.1/2^-^ CD44^hi^ CD27^-^ γδ T cells, myeloid cells (CD11b^+^), and CD4 and CD8 T cells was performed. Consistent with our *in vitro* observations, Vγ1.1/2^-^ CD44^hi^ CD27^-^ γδ T cells and myeloid cells contained translocated Yop *in vivo* (Fig 6G). *Y*. *pseudotuberculosis* was relatively inefficient at Yop translocation into CD4 and CD8 T cells (Figs 6G and S7C). Collectively, these data show that foodborne YopJ^C172A^ *Y*. *pseudotuberculosis* infection of naïve mice elicits IFNγ production in adaptive γδ T cells and that Yop can be translocated into adaptive Vγ4 T cells in vivo.

## Discussion

In this study, we assessed the subversion of an adaptive subset of γδ T cells specialized in the promotion of pathogen resistance at the intestinal mucosa through the production of anti-infective cytokines like IFNγ and IL-17A [37]. While limited evidence suggests that Yop effectors directly target T cells to subvert their function, we identified that the *Y*. *pseudotuberculosis* effector molecule YopJ directly inhibits IFNγ production from adaptive CD44^hi^ CD27^-^ γδ T cells to subvert host immunity in mice. Additionally, we demonstrate that circulating human Vδ2^+^ T cells are similarly inhibited by direct translocation of YopJ, demonstrating that the direct targeting of γδ T cells by *Y*. *pseudotuberculosis* to inhibit IFNγ production is a conserved pathway of immune evasion in humans. Thus, *Y*. *pseudotuberculosis* Yop effectors translocated into murine Vγ4 T cells and human Vδ2^+^ T cells directly subvert their anti-microbial functions and host immunity by limiting IFNγ production.

While *Yersinia* mediated inhibition of conventional T cells has been previously reported, studies have largely focused on the indirect subversion of T cells that is mediated by translocation of Yop effectors into myeloid cells [55,82]. For example, YopJ/P appears to primarily subvert conventional T cell function through indirect mechanisms associated with inhibiting DC [22,26]. On the contrary, the study of γδ T cells in the context of *Y*. *pseudotuberculosis* infection has been primarily limited to the potential antigens that drive γδ T cell recognition of infection [84–89].

After phagocytosis of pathogens, activated DC migrate to lymph nodes and present antigen to T cells. APC-derived IL-12 further shapes T cell responses by providing a critical signal during T cell activation [90]. Interestingly, *Y*. *pestis* can limit both the migratory capacity of DC and the production of IL-12 [91], and *Y*. *enterocolitica* can induce programmed death of DC and inhibit antigen presentation [22]. *Y*. *pseudotuberculosis* YopJ can also indirectly limit NK cell function by interfering with DC TLR4 signaling pathways and YopP in *Y*. *enterocolitica* can limit NK cell function through STAT4 inhibition [21,92]. Given that IL-12 signaling promotes STAT4 phosphorylation and IFNγ production in γδ T cells [29,93] and *Yersinia spp*. can inhibit DC, a potential extrinsic mechanism emerges for *Y*. *pseudotuberculosis* to inhibit γδ T cell responses by suppressing DC functions. In line with these observations, IL-12p40 was critical for the induction of phospho-STAT4 in Vγ4 T cells after stimulation with *Y*. *pseudotuberculosis* in *in vitro* cultures. In contrast, *Y*. *pseudotuberculosis* and IL-12 were unable to directly elicit IFNγ production from a highly enriched population of *in vitro* expanded γδ T cells suggesting that IL-12 is required but not sufficient for Vγ4 T cell IFNγ production. However, stimulation of MLN cell suspensions with ΔYopB or YopJ^C127A^ *Y*. *pseudotuberculosis* elicited STAT4 phosphorylation among Vγ4 T cells suggesting that translocation of Yop effectors and YopJ in particular subverts Vγ4 T cell function. Tracking Yop translocation *in vitro* revealed that Vγ4 T cells that contain Yop had reduced pSTAT4 levels and inhibited IFNγ production. Importantly, Vγ4 T cells that did not contain Yop effectors from the same cultures expressed higher pSTAT4 levels and comparable IFNγ production as Vγ4 T cells stimulated with ΔYopB-βla *Yptb*. Additionally, IL-12 levels were comparable between cultures stimulated with WT and YopJ^C172A^ *Y*. *pseudotuberculosis*. Collectively, these data suggest that functional impairment of Vγ4 T cells was mediated by direct translocation of YopJ into Vγ4 T cells and cell intrinsic mechanisms *in vitro*.

The YopJ effector family has been increasingly described by their acetyltransferase function on serine, threonine, and lysine amino acid residues [19,94]. Serine and threonine are common targets of phosphorylation to propagate signaling cascades or elicit functional consequences. For example, phosphorylation of STAT4 leads to dimerization and transport to the nucleus to promote transcription of STAT4 target genes. Acetylation of these target residues may inhibit phosphorylation and downstream signaling events [70]. Thus, a potential mechanism of YopJ subversion of Vγ4 T cells is through acetylation of STAT4 to inhibit the phosphorylation or dimerization of STAT4. Other potential targets of YopJ acetyltransferase activity are the IL-12R and Janus kinases upstream of STAT4 activation. YopJ has also been reported to have cysteine protease, lysine acetyltransferase, ubiquitin-like protein protease, and deubiquitinase activity that may provide other potential avenues for YopJ to modulate the function of Vγ4 T cells through STAT4 [94–96].

An important aspect of *Y*. *pseudotuberculosis* pathogenesis unveiled by this work is the preferential targeting of a specialized subset of γδ T cells for delivery of inhibitory Yop effector molecules. *Y*. *pseudotuberculosis* injected Yop effectors into adaptive γδ T cells in a similar proportion as macrophages and DC and to a much greater extent than conventional CD4 or CD8 T cells. Of note, *Y*. *pseudotuberculosis* has been reported to translocate Yop effectors more efficiently into T_reg_ cells than conventional CD4 T cells at high MOI to modulate their function [65]. The preferential targeting of Vγ4 T cells in this system is associated with expression of the β_1_-integrin by adaptive Vγ4 T cells. Additionally, the majority of adaptive Vγ4 T cells are anatomically segregated from conventional T cells in the paracortex by their localization in the interfollicular and medullary areas of the gut draining lymph nodes [38]. The distinct localization of adaptive Vγ4 T cells may facilitate interactions with *Y*. *pseudotuberculosis in vivo*. Loss of the adhesin YadA but not Inv abrogated YopJ mediated γδ T cell inhibition, suggesting that *Y*. *pseudotuberculosis* utilizes YadA to target adaptive γδ T cells for Yop translocation, consistent with previous studies suggesting that the adhesin Inv is largely dispensable for *Y*. *enterocolitica* virulence [61,97]. Interestingly, this appears distinct from the targeting of conventional CD4 T cells that relies on Inv and is enhanced in the absence of YadA [11,65].

While our data demonstrates that STAT4 phosphorylation is inhibited by YopJ, our RNA-Seq analysis suggests that other targets may also be affected. One of YopJ’s known targets is the MAPK family of proteins that can have broad effects on cell proliferation, differentiation, survival, and apoptosis [18]. The MAPK pathway in CD4 T cells and NK cells may also promote STAT4 activity and downstream IFNγ mRNA stabilization, respectively [98,99]. Our data demonstrate a broad impact of YopJ on adaptive γδ T cell proliferation, metabolism, cell cycle, RNA/DNA processing, and ER/Golgi processing gene expression networks. These pathways may be regulated by MAPK family member activity [100–105]. Homer motif analysis identified other potential means by which YopJ may regulate IFNγ production. For example, Ets-1 is a T-bet cofactor and necessary for Th1 IFNγ responses [106]. Increases in ETS-domain family of transcription factor motifs were associated with type 3 innate lymphoid cells (ILC3) but not Th17 cells [107], which may suggest that one of the potential mechanisms of YopJ mediated inhibition may target conserved pathways in unconventional lymphocyte populations. Many NK cell receptors are also expressed on γδ T cells and may facilitate TCR independent effector functions [108–110]. In line with this, our profiling demonstrates that YopJ limits gene expression of *Nkg7*, which has recently been reported to promote cytotoxic granule release and inflammation during infection and cancer [72], and *Prf1*, which encodes the pore forming molecule perforin necessary to deliver lytic machinery into target cells [111]. Finally, interactions with *Y*. *pseudotuberculosis* YopJ led to the upregulation of *Ulbp1*, which encodes a stress-induced NKG2D ligand, and *Idi1*, which encodes an enzyme in the mevalonate pathway. As human γδ T cells respond to phospho-antigens derived from the non-mevalonate pathway in bacteria and mammalian mevalonate pathway in humans [112], this may limit the removal of translocated cells through NK or γδ T cell sensing mechanisms and suggests a broad mechanism to subvert human immunity. Thus, our findings suggest that adaptive Vγ4 T cells provide dynamic anti-infectious immunity that is subverted by direct translocation of YopJ.

A number of studies have highlighted the importance of IFNγ production from conventional CD4 and CD8 T cells, NK cells, and ILC3 for *Yersinia* resistance [40,43,113,114], although the *in vivo* relevance of Yop inhibition of conventional T cells has not been addressed. Foodborne infection with YopJ^C172A^ *Y*. *pseudotuberculosis* led to an enhanced response from Vγ4 T cells, including increased IFNγ production, that was associated with a more rapid recovery of weight. As IL-12 was critical for Vγ4 T cell derived IFNγ production *in vitro*, the role of IL-12 after foodborne infection of Balb/c mice was assessed. Consistent with previous studies [22,40,115,116], IL-12 appeared critical for protection against foodborne WT and YopJ^C172A^ *Y*. *pseudotuberculosis* infection. As serum IL-12 was only readily detectable after YopJ^C172A^ *Y*. *pseudotuberculosis* infection, increased IL-12 may also contribute to the enhanced IFNγ response from Vγ4 T cells *in vivo*. Finally, assessment of cell populations targeted for Yop translocation *in vivo* was comparable to the results from the *ex vivo* MLN cultures. The highest percentage of Yop^+^ cells were among CD11b^+^ cells and Vγ4 T cells. Given the low MOI used in our *ex vivo* studies with the WT *Yptb*-βla reporter and the lack of inhibition observed in Vγ4 T cells that lack Yop effectors from the same culture conditions, it is likely that intrinsic Yop effects on Vγ4 T cells is a mechanism of inhibiting Vγ4 T cell function *in vivo*. Thus, while *Y*. *pseudotuberculosis* can use γδ T cell intrinsic mechanisms to subvert the γδ T cell IFNγ response, multiple mechanisms may be available for *Yersinia spp*. to subvert γδ T cell functions to aid pathogenesis *in vivo*. As we previously demonstrated that foodborne but not i.v. infection led to adaptive Vγ4 T cell responses [37], our physiologic foodborne *Y*. *pseudotuberculosis* infection model revealed novel aspects of *Yersinia* pathogenesis and adaptive Vγ4 T cell biology.

In summary, the *Y*. *pseudotuberculosis* effector YopJ directly inhibits essential anti-effective functions of murine Vγ4 T cells and human Vδ2^+^ T cells. *Y*. *pseudotuberculosis* targeted Vγ4 T cells in a T3SS- and YadA-dependent process to deliver Yop effectors directly into Vγ4 T cells. *Ex vivo* whole tissue cultures revealed that direct inhibition of Vγ4 T cell function was the major mechanism of Vγ4 T cell subversion. YopJ translocation led to a dramatic reduction in STAT4 phosphorylation levels and IFNγ production, which is important for protection from *Yersinia*. YopJ also inhibited a broad anti-infective gene signature. Thus, these findings add substantial insight into YopJ effector functions on murine and human γδ T cells and the pathogenesis of foodborne *Y*. *pseudotuberculosis* infection.

## Materials and methods

### Ethics statement

All animal experiments were conducted in accordance with the Stony Brook University Institutional Animal Care and Use Committee and National Institutes of Health guidelines. Blood collection from healthy human donors was approved by the Institutional Review Board at Stony Brook University.

### Mice

Female 8–12 week old BALB/cJ mice were purchased from the Jackson Laboratory. Mice were euthanized by CO_2_ inhalation.

### Human studies

Blood was sampled from a total of 6 adult healthy human donors of either gender between the ages of 20 and 40. Studies were designed so no randomization to experimental groups was necessary. Donors provided written informed consent.

### Bacteria

Bacteria strains used in this study include: *Y*. *pseudotuberculosis* on the 32777 background WT strain, WT32777c, YopJ^C172A^, YopH^R409A^, ΔYopB, YopE^R144A^, YopT^C139A^, ΔYopM, ΔYpkA, ΔYopK, WT *Yptb*-βla, and ΔYopB *Yptb*-βla. *Y*. *pseudotuberculosis* on the IP2666 background WT strain, ΔYopB, ΔInv, ΔYadA, ΔInv ΔYadA, and ΔYopB ΔInv ΔYadA. See Table 1 for details. All strains were stored in 25% glycerol stocks at -80°C. For stimulations, *Y*. *pseudotuberculosis* strains were cultured overnight at 28°C and 220 RPM in LB media. The following morning, *Y*. *pseudotuberculosis* was sub-cultured 1:10 in LB and 50 mM CaCl_2_ at 37°C and 220 RPM for approximately 2 hours. Stimulation doses were based on the OD600.

### Foodborne *L*. *monocytogenes* immunization

*L*. *monocytogenes* (EGDe strain) expressing a mutation in the internalin A gene (InlA^M^) was used for foodborne infection to facilitate epithelial cell invasion [117]. InlA^M^ *L*. *monocytogenes* was cultured overnight at 37°C and 220 RPM in BHI media. The following morning, *L*. *monocytogenes* was sub-cultured 1:10 in BHI at 37°C and 220 RPM for approximately 2 hours. Infection doses were based on the OD600. Mice were food and water deprived for 4 hours. Approximately 0.5 cm^3^ bread pieces were inoculated with 2x10^9^ CFU *L*. *monocytogenes* in 50 μL. Mice were monitored to ensure the inoculated bread was consumed within 1 hour. Mice that did not fully consume bread were removed from the study. Bacterial infection doses were confirmed by plating inoculum on BHI.

### Foodborne *Y*. *pseudotuberculosis* infection

*Y*. *pseudotuberculosis* strains (Table 1) were cultured overnight at 28°C and 220 RPM in LB media. Infection doses were based on the OD600. Mice were food and water deprived for 4 hours. Approximately 0.5 cm^3^ bread pieces were inoculated with 2-4x10^7^ CFU for WT32777 *Y*. *pseudotuberculosis*, 2-4x10^7^–2-4x10^8^ CFU for YopJ^C172A^ *Y*. *pseudotuberculosis*_,_ or 2x10^9^ CFU for WT *Yptb*-βla infection in 50 μL. Mice were monitored to ensure the inoculated bread was consumed within 1 hour. Mice that did not fully consume bread were removed from the study. Bacterial infection doses were confirmed by plating inoculum on LB.

### Single cell preparations, *Y*. *pseudotuberculosis* stimulations, and flow cytometry

MLN from *L*. *monocytogenes* immunized mice were harvested 9 days after immunization and mechanically dissociated using a syringe plunger through a 70 μm cell strainer into a single cell suspension. Cells were resuspended in IMDM (Gibco) supplemented with 10% fetal bovine serum, 0.01 M HEPES, 100 μM non-essential amino acids (Gibco), 2 mM L-alanyl-L-glutamine dipeptide in 0.85% NaCl or 1x Glutamax (Gibco), and 1 mM sodium pyruvate. Cells were counted using a Vi-CELL Viability Analyzer (Beckman Coulter). Cells were stimulated in 96 well round-bottom tissue culture treated plates with various strains of *Y*. *pseudotuberculosis* at 1 or 10 MOI (1 MOI for WT or ΔYopB *Yptb*-βla and 10 MOI for all other *Y*. *pseudotuberculosis* stimulations, unless otherwise indicated) at 37°C/5% CO_2_. 100 U/mL of penicillin and 100 μg/mL of streptomycin were added to cells 2 hours post-stimulation. Cells were stimulated for a total of 24 hours or as indicated. BD GolgiPlug (BD Biosciences) was added 5 hours prior to the end of stimulation. If translocation was assessed, β-lactamase Loading Solutions kit (Invitrogen) was used to load CCF4-AM by incubating CCF4-AM at RT with cells for 1 hour in the dark. Cells were then processed for surface staining via incubation with live/dead stain, antibody, and Fc block (BioXcell) for 20 min in the dark at 4°C. Antibodies used included antibodies specific to CD45, CD3, TCRδ, CD8, CD4, Vγ1.1, Vγ2, CD44, CD27, F4/80, CD11b, MHCII, CD11c, and CD29 (BioLegend). Cells were fixed, permeabilized, and stained with anti-IFNγ, anti-IRF8, or anti-STAT4 using BD Cytofix/Cytoperm kit (BD Biosciences) for intracellular cytokine staining. Functional γδ T cell analysis was done by stimulation with BD leukocyte activation cocktail (containing PMA, ionomycin, and brefeldin A; BD Pharmingen) for 4 hours prior to staining. Flow cytometry data were acquired using a BD LSRFortessa and analyzed by FlowJo software (BD Biosciences). Cell culture supernatant was analyzed for IL-12p70 using the BioLegend ELISA MAX Deluxe Set Mouse IL-12 (p70) kit per manufacturer instructions.

### Human γδ T cell response

Blood was drawn and collected from healthy human donors in BD Vacutainer sodium heparin tubes (BD Biosciences). Blood was diluted 1:1 with 1x PBS at room temperature. Peripheral blood mononuclear cells (PBMC) were isolated from the buffy coat of Ficoll-paque PLUS gradient centrifugation (GE Healthcare) for 20 min at 1,400 × g without a brake. PBMC were washed with 1x PBS at room temperature and resuspended in IMDM supplemented with 10% fetal bovine serum, 0.01 M HEPES, 100 μM Non-essential amino acids (Gibco), 2 mM L-alanyl-L-glutamine dipeptide in 0.85% NaCl or 1x Glutamax (Gibco), and 1 mM sodium pyruvate. *Y*. *pseudotuberculosis* strains were cultured overnight at 28°C and 220 RPM in LB media the night prior. The following morning, *Y*. *pseudotuberculosis* was sub-cultured 1:10 in LB and 50 mM CaCl_2_ at 37°C and 220 RPM for approximately 2 hours. Stimulation doses were based on the OD600. Cells were counted using a Vi-CELL Viability Analyzer (Beckman Coulter). Cells were stimulated in 96 round-bottom tissue culture treated plates with various strains of *Y*. *pseudotuberculosis* at 1 MOI at 37°C/5% CO_2_. 1x penicillin and streptomycin (100 U/mL penicillin and 100 μg/mL streptomycin) were added to cells 2 hours post-stimulation. Cells were stimulated for a total of 24 hours or as indicated. BD GolgiPlug (BD Biosciences) was added 5 hours prior to the end of stimulation. If translocation was assessed, β-lactamase Loading Solutions kit (Invitrogen) was used to load CCF4-AM by incubating CCF4-AM at RT with cells for 1 hour in the dark. Cells were then processed for surface staining via incubation with live/dead stain, antibody, and Fc block (BioXcell) for 20 min in the dark at 4°C. Antibodies used included antibodies specific to Vδ2, CD3, TCRδ, (BioLegend). Cells were fixed, permeabilized, and stained with anti-IFNγ using BD Cytofix/Cytoperm kit (BD Biosciences) for intracellular cytokine staining. Flow cytometry data were acquired using a BD LSRFortessa and analyzed by FlowJo software (BD Biosciences).

### Phospho-flow cytometry

After surface staining for flow cytometry, cells were washed and stained for pSTAT4 using a methanol-based approach. Cells were fixed in 4% PFA/1.5% methanol for 30 minutes in the dark at 4°C. Cells were then washed and incubated in methanol in the dark at 20°C for 45 minutes. After washing, cells were stained with anti-pSTAT4 (Y693)-PE (BD Biosciences) and washed once more. Flow cytometry data were acquired using a BD LSRFortessa and analyzed by FlowJo software (BD Biosciences).

### Enumeration of *Y*. *pseudotuberculosis* burden

MLN were crushed and diluted in media prior to plating on LB agar. Total *Y*. *pseudotuberculosis* burden per organ was calculated.

### Sequencing and analysis

Samples were prepared after *Y*. *pseudotuberculosis* stimulation as described above. Cell preparations were stimulated with 10 MOI of WT or YopJ^C172A^ *Y*. *pseudotuberculosis* or 1 MOI of WT *Yptb*-βla for 24 hours. 500 Vγ1.1/2^-^ CD44^hi^ CD27^-^ γδ T cells were flow sorted directly into a tube with NEBNext Cell Lysis Buffer and Murine RNase Inhibitor and processed for RNA sequencing using NEBNext Single Cell/Low Input RNA Library Prep Kit (Illumina). Sequencing was performed at the Cold Spring Harbor Laboratory sequencing core on a NextSeq500. Fastq files were produced as an output of the sequencing files. Fastq were run through FastQC to perform quality control of transcripts prior to alignment. Fastq files were pair-ended aligned to GRCm38/mm10 by way of HISAT2 and output as .BAM files [118]. Raw counts of aligned transcripts were quantified with FeatureCounts [119]. Dimensionality reduction was performed with PCA analysis with the axes PC1 and PC2 in R-studio [120]. To determine differential expression between samples, FeatureCounts raw count matrix was analyzed through DESeq2 with a parametric fitting normalized to the geometric mean of each individual gene across samples [121]. Cutoff values for significance and quality control were a p-value of <0.05 and FDR-value of <0.10, respectively. Significantly differentially expressed genes were visualized on a heatmap with a dendrogram that was clustered through average linkage. The distance measurement on the dendrogram used was through the Euclidean method. Overlapping expressions between gene differential expression sets were filtered with R-studio. Upregulated and downregulated genes from the differential expression analysis were separated with R-studio and these Gene IDs were used for HOMER motif analysis [74]. Parameters of analysis of each gene used were 400bp preceding the initiation site and 100bp after the initiation site. The length of the motifs analyzed was set between 8 and 10.

### γδ T cell purification

γδ T cells were expanded *in vitro* according to published protocols [122]. The MLN and spleen were isolated and processed into a single cell suspension 9 days post foodborne *L*. *monocytogenes* infection of Balb/c mice. Red blood cells were lysed with red blood cell lysis buffer or ammonium chloride for 1 minute and cells from the MLN and spleen were combined. γδ T cells were enriched by negative selection using the following rat IgG primary antibodies from BioLegend: anti-CD4 (clone GK1.5), anti-CD8α (clone 53–6.7), anti-B220 (clone RA3-6B2), anti-MHC-II (clone M5/114.15.2), and anti-CD11b (clone M1/70). The MACS goat anti-rat IgG kit (Miltenyi Biotec) was used per manufacturer instructions with MACS LD columns and a QuadroMACS magnet. Enriched cells were cultured in 48-well plates coated overnight with 5 ug/ml anti-TCRδ (clone GL3). Cells were cultured in RPMI 1640 supplemented with 25 mM HEPES (Gibco), 1x glucose (Gibco), 10 g/ml folate (Sigma Aldrich), 1x sodium pyruvate (Gibco), 5x10^5^ M 2 beta-mercaptoethanol (Sigma Aldrich), 1x Glutamax (Gibco), 1x penicillin-streptomycin (Gibco), and 10% FBS with 100 U/ml recombinant human IL-2. After 2 days of culture, cells were transferred into new wells with the same culture media to rest for 5 days. Cells were then stimulated with 10 MOI YopJ^C172A^ *Y*. *pseudotuberculosis* with 0.1, 1, or 10 ng/ml of recombinant murine IL-12p70 (Peprotech).

### *In vivo* anti-IL-12p40 antibody treatment and serum collection

On the day of foodborne WT or YopJ^C172A^ *Y*. *pseudotuberculosis* infection and on day 2, 4, and 6 after infection, mice were treated with 0.2 mg of anti-IL-12p40 (clone C17.8; BioLegend) by i.p. injection. Blood was collected via tail vein on day 2, 4, and 6 after infection. Blood was incubated at ambient temperature for 30 minutes before being spun down at 1500G for 10 minutes at 4°C. Serum was isolated and analyzed for IL-12p70 with the BioLegend ELISA MAX Deluxe Set Mouse IL-12 (p70) kit.

### *Ex vivo* anti-IL-12p40 and recombinant IL-12p70 treatments

At the start of *Y*. *pseudotuberculosis* stimulation of MLN cell suspensions, cultures were treated with 10 μg/ml anti-IL-12p40 (clone C17.8; BioLegend) for neutralization. In other conditions, recombinant murine IL-12p70 (Peprotech) was added at the start of *Y*. *pseudotuberculosis* stimulation of MLN cell suspensions at 2, 10, or 50 ng/ml.

### Statistical analysis

GraphPad Prism 6 software (GraphPad Software Inc.) was used for statistical analysis. The differences between the means were compared using the statistical analysis described in the associated figure legends. All the data are presented as mean ± SEM and *p* < 0.05 was considered significant. *p < 0.05, **p < 0.01, ***p < 0.001, ****p < 0.0001.

## Supporting information

S1 Data Excel spreadsheet containing the underlying numerical data for Figs 1A–1C, 2A and 2B, 3A, 5A–5D and 6A–6F. (XLSX)

S1 Fig Use of the *Yptb*-βla reporter to track Yop translocation. (**A**) MLN suspensions from *L*. *monocytogenes* infected mice were stimulated with WT or ΔYopB *Y*. *pseudotuberculosis* containing a β-lactamase translocation reporter (*Yptb*-βla) for 2 hours and given antibiotics. Cells were loaded with CCF4-AM dye to measure β-lactamase activity. FITC indicates CCF4-AM loaded cells without translocation (Yop^-^) and BV421 indicates CCF4-AM loaded cells with Yop translocation (Yop^+^). Vγ1.1/2^-^ CD44^hi^ CD27^-^ γδ T cells were analyzed for Yop translocation 2 hours post stimulation at an MOI of 10. Representative contour plots are displayed. (**B**) MLN from *L*. *monocytogenes* infected mice were stimulated with *Yptb*-βla for 2 hours and given antibiotics. Yop translocation was detected as described above. The indicated cell populations were analyzed for Yop translocation 2 hours after stimulation. Representative contour plots are displayed. (**C**) MLN from *L*. *monocytogenes* infected mice were stimulated with *Yptb*-βla for 2 hours and given antibiotics. Vγ1.1/2^-^ CD44^hi^ CD27^-^ γδ T cells were analyzed for Yop translocation 2 hours post stimulation at the indicated MOI and quantified for Yop translocation. Data consists of one experiment with 2–10 mice/group and the graphs depict the mean ± SEM in (**A**-**C**). ****p < 0.0001, ***p < 0.001, and **p < 0.01. A t-test was used for (**A**), and a repeated measures one-way ANOVA was used for (**C**). Comparisons were performed to ΔYopB *Y*. *pseudotuberculosis* in (**A**) and as depicted in (**C**).
(TIF)

S2 Fig The majority of Vγ1.1/2^-^ CD44^hi^ CD27^-^ γδ T cells and γδ T cells containing Yop express β1-integrin. (**A**) MLN from *L*. *monocytogenes* infected mice were isolated and processed into single cell suspensions. Vγ1.1/2^-^ CD44^hi^ CD27^-^ γδ T cells, CD4 T cells, and CD8 T cells were analyzed for β_1_-integrin expression. (**B**) MLN suspensions from *L*. *monocytogenes* infected mice were loaded with CCF4-AM dye and stimulated with 10 MOI WT *Y*. *pseudotuberculosis* containing a β-lactamase translocation reporter. CCF4-AM dye reports the occurrence of β-lactamase activity and Yop translocation. γδ T cells that contain Yop (Yop^+^) or do not contain Yop (Yop^-^) were analyzed for β1-integrin expression 2 hours after stimulation. Representative histogram plots are displayed. Data is pooled from two experiments with a total of 7 mice/group and the graphs depict the mean ± SEM in (**A**-**C**). ****p < 0.0001 and ***p < 0.001. A repeated measures one-way ANOVA was used for (**A**) and a t-test was used for (**B**), and. Comparisons were done to adaptive γδ T cells in (**A**) and as depicted in (**B**).
(TIF)

S3 Fig YopJ regulates the transcriptional profile of Vγ1.1/2^-^ CD44^hi^ CD27^-^ γδ T cells. (**A** and **B**) MLN from *L*. *monocytogenes* infected mice were stimulated with 10 MOI of WT *Y*. *pseudotuberculosis* (WT) or mutant YopJ *Y*. *pseudotuberculosis* (YopJ^C172A^) for 24 hours. Antibiotics were given 2 hours post-stimulation. Five hundred Vγ1.1/2^-^ CD44^hi^ CD27^-^ γδ T cells from each stimulation were flow sorted and processed for RNA sequencing. The heat map depicts upregulated genes in Vγ1.1/2^-^ CD44^hi^ CD27^-^ γδ T cells after YopJ^C172A^ *Y*. *pseudotuberculosis* stimulation and individual genes are listed. (**C**-**E**) Genes differentially expressed (downregulated) that overlapped between RNA sequencing analyses as displayed in the Venn diagram in (**C**) to select for direct effects of YopJ on Vγ1.1/2^-^ CD44^hi^ CD27^-^ γδ T cells. The heat map depicts downregulated genes in Vγ1.1/2^-^ CD44^hi^ CD27^-^ γδ T cells from the analysis in (**C**). Individual genes are listed in (**E**). Each experiment was performed once with biologic replicates. The cutoff for gene significance was p < 0.05 and FDR < 0.10.
(TIF)

S4 Fig YopJ does not inhibit IL-17A production in Vγ1.1/2^-^ CD44^hi^ CD27^-^ γδ T cells. MLN cell suspensions from *L*. *monocytogenes* infected mice were stimulated with 10 MOI of WT, YopJ^C172A^, or ΔYopB *Y*. *pseudotuberculosis* for 24 hours. Antibiotics were given 2 hours after stimulation and brefeldin A was added for the last 5–6 hours. Vγ1.1/2^-^ CD44^hi^ CD27^-^ γδ T cells were analyzed for IL-17A production after stimulation. Representative histograms are displayed. The graph depicts mean ± SEM and represents two independent experiments with 4 mice per group. A repeated measures one-way ANOVA was used. * p < 0.05.
(TIF)

S5 Fig YopJ impacts IFNγ related transcription factor motifs but not STAT4 protein. (**A**) Homer motif analysis was performed on the RNA sequencing results for the YopJ^C172A^ and WT *Y*. *pseudotuberculosis* comparison from Fig 5. The panel highlights the top transcription factor motifs of the ETS, SP/KLF, and IRF family of proteins identified in YopJ^C172A^ stimulated Vγ1.1/2^-^ CD44^hi^ CD27^-^ γδ T cells. (**B**) MLN from *L*. *monocytogenes* infected mice were stimulated with 10 MOI of WT, YopJ^C172A^, or ΔYopB *Y*. *pseudotuberculosis* for 6 hours. Antibiotics were given 2 hours post-stimulation. Vγ1.1/2^-^ CD44^hi^ CD27^-^ γδ T cells were analyzed for IRF8 levels with or without anti-IL12p40 neutralizing antibody. The graph depicts the percentage of IRF8 protein expression among Vγ1.1/2^-^ CD44^hi^ CD27^-^ γδ T cells after WT, YopJ^C172A^, or ΔYopB *Y*. *pseudotuberculosis* stimulation. Data depict two pooled experiments with a total of 8 mice/group and represents the mean ± SEM. (**C**) The genes from the RNAseq and Homer motif analysis in Figs 4F and 4G and S3B and S5A were compared to an existing STAT4 ChIP-on-chip dataset to identify common genes. Genes from our dataset that were represented in the top 1000 genes of the Chip-on-chip dataset are displayed. (**D**) STAT4 KO spleens are shown in maroon and WT spleens are shown in black in representative histograms. The graph depicts the MFI of STAT4 protein expression in bulk γδ T cells. Data depicts one experiment with 4 mice/group and represents the mean ± SEM. ****p < 0.0001, ***p < 0.001, **p < 0.01, *p < 0.05. A repeated measures one-way ANOVA was used for (**B**), and a t-test was used for (**D**). Comparisons were performed as depicted in (**B**) and to Naïve WT in (**D**).
(TIF)

S6 Fig IL-12 is insufficient to induce IFNγ and does not readily overcome the inhibition of YopJ. (**A**) γδ T cells enriched from the MLN and spleen of *L*. *monocytogenes* infected mice were expanded with plate bound γδTCR antibody for 2 days and rested for 5 days. After expansion, ~ 50% of cells were γδ T cells, and the majority of those were Vγ4 T cells. The enrichment summary reflects the mean enrichment from 4 samples. Afterwards, γδ T cells were isolated from cultures and stimulated with YopJ^C172A^ *Y*. *pseudotuberculosis* with 0.1, 1, or 10 ng/ml IL-12p70 for 24 hours. Antibiotics were added 2 hours after stimulation and brefeldin A was added for the last 5–6 hours. Histograms display IFNγ production from Vγ1.1/2^-^ CD44^hi^ CD27^-^ γδ T cells under different culture conditions. Data depicts one experiment with 4 mice pooled and split into the indicated stimulation conditions. (**B**) MLN cell suspensions from *L*. *monocytogenes* infected mice were stimulated with 10 MOI of WT *Y*. *pseudotuberculosis* in the presence of 2, 10, or 50 ng/ml IL-12p70 or 10 MOI of YopJ^C172A^ *Y*. *pseudotuberculosis* for 24 hours. Antibiotics were given 2 hours after stimulation and brefeldin A was added for the last 5–6 hours. Vγ1.1/2^-^ CD44^hi^ CD27^-^ γδ T cells were analyzed for IFNγ production. Representative histograms of IFNγ production from Vγ1.1/2^-^ CD44^hi^ CD27^-^ γδ T cells are displayed. The graph depicts mean ± SEM from one experiment with 4 mice per group *p < 0.05. A repeated measures one-way ANOVA was used for comparisons to YopJ^C172A^ *Y*. *pseudotuberculosis* in (**B**).
(TIF)

S7 Fig The impact of foodborne infection of mice with *Y*. *pseudotuberculosis*. (**A**) Balb/c mice were foodborne infected with the indicated doses of WT or mutant YopJ^C172A^ *Y*. *pseudotuberculosis* and tissues were analyzed 9 days post-infection. Bacteria burden was quantified from the MLN. Data reflect 3–5 mice per group pooled from 3 independent experiments and the graphs depict the mean ± SEM. (**B**) Balb/c mice were foodborne infected with the indicated doses of WT or mutant YopJ^C172A^ *Y*. *pseudotuberculosis*. Nine days post infection, MLN suspensions from WT or YopJ^C172A^ *Y*. *pseudotuberculosis* infected mice were stimulated with PMA/ionomycin and brefeldin A for 4 hours. Vγ1.1/2^-^ CD44^hi^ CD27^-^ γδ T cells were analyzed for IFNγ production. Representative histograms are displayed and quantified. Data depicts one experiment with 3 mice per group. (**C**) Balb/c mice were foodborne infected with WT (2-4x10^7^ CFU) or YopJ^C172A^ *Y*. *pseudotuberculosis* (2-4x10^8^ CFU) and treated with 0.2 mg/mouse of anti-IL12p40 on days 0, 2, 4, and 6 post infection. IL-12p70 concentrations were determined from serum at days 2, 4, and 6 post infection. Data represent 2 independent experiments with a total of 9 mice per group. Serum samples were pooled into groups of 3 per experimental condition. (**D**) Balb/c mice were foodborne infected with 2x10^9^ CFU *L*. *monocytogenes* to elicit a Vγ1.1/2^-^ CD44^hi^ CD27^-^ γδ T cell population *in vivo*. 30 days post infection, adaptive Vγ1.1/2^-^ CD44^hi^ CD27^-^ γδ T cells from the MLN of immune mice were analyzed for Yop translocation using the CCF4-AM assay. Representative contour plots are shown. Yop translocation (Yop^+^) among the indicated populations represents background staining as a negative control for Fig 6F. The graph depicts the mean ± SEM and is pooled from 2 experiments with a total of 4 mice per group. ****p < 0.0001, *p < 0.05. A one-way ANOVA was used for (**A**), and an unpaired t-test was used for (**B**). Comparisons were performed to uninfected in (**A**) and to 5x10^7^ WT *Y*. *pseudotuberculosis* in (**B**).
(TIF)
