# Correction: γδ T cell IFNγ production is directly subverted by *Yersinia pseudotuberculosis* outer protein YopJ in mice and humans

**DOI:** 10.1371/journal.ppat.1010586

**Published:** 2022-05-25

**Authors:** Timothy H. Chu, Camille Khairallah, Jason Shieh, Rhea Cho, Zhijuan Qiu, Yue Zhang, Onur Eskiocak, David G. Thanassi, Mark H. Kaplan, Semir Beyaz, Vincent W. Yang, James B. Bliska, Brian S. Sheridan

There are errors in figure references in the subsection Foodborne infection with YopJ^C172A^ *Y*. *pseudotuberculosis* induces IFNγ production in adaptive Vγ4 T cells of the Results.

There is an error in the second-to-last sentence of the first paragraph. The correct sentence is: IL-12p70 was detectable 6 days after YopJ^C172A^ *Y*. *pseudotuberculosis* infection but was mostly below the limit of detection after WT *Y*. *pseudotuberculosis* infection (S7C Fig).

There is an error in the fifth sentence of the second paragraph. The correct sentence is: Indeed, infected mice contained detectable *Y*. *pseudotuberculosis* in the MLN 3 days after foodborne infection (Fig 6E).

There are errors in the seventh and eighth sentences of the second paragraph. The correct sentences are: Consistent with our *in vitro* observations, Vγ1.1/2^-^ CD44^hi^ CD27^-^ γδ T cells and myeloid cells contained translocated Yop *in vivo* (Fig 6F). *Y*. *pseudotuberculosis* was relatively inefficient at Yop translocation into CD4 and CD8 T cells (Figs 6F and S7D).
