# Supplementary figures and images for: γδ T cell IFNγ production is directly subverted by Yersinia pseudotuberculosis outer protein YopJ in mice and humans
Source: PLoS Pathog. 2021 Dec 6;17(12):e1010103. doi: 10.1371/journal.ppat.1010103 (PMC8648121; doi:10.1371/journal.ppat.1010103)

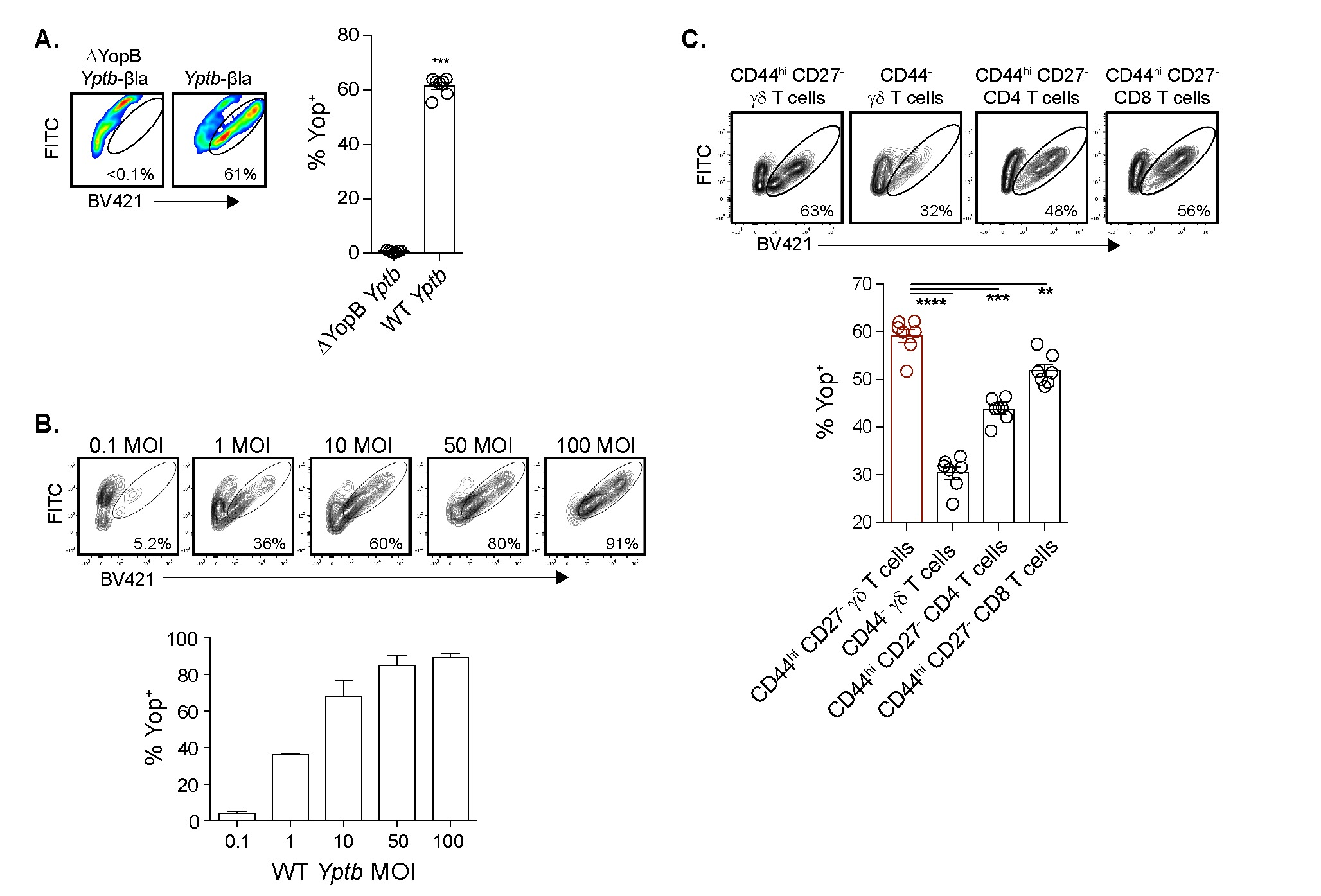

Supplement: S1 Fig — (A) MLN suspensions from L. monocytogenes infected mice were stimulated with WT or ΔYopB Y. pseudotuberculosis containing a β-lactamase translocation reporter (Yptb-βla) for 2 hours and given antibiotics. Cells were loaded with CCF4-AM dye to measure β-lactamase activity. FITC indicates CCF4-AM loaded cells without translocation (Yop-) and BV421 indicates CCF4-AM loaded cells with Yop translocation (Yop+). Vγ1.1/2- CD44hi CD27- γδ T cells were analyzed for Yop translocation 2 hours post stimulation at an MOI of 10. Representative contour plots are displayed. (B) MLN from L. monocytogenes infected mice were stimulated with Yptb-βla for 2 hours and given antibiotics. Yop translocation was detected as described above. The indicated cell populations were analyzed for Yop translocation 2 hours after stimulation. Representative contour plots are displayed. (C) MLN from L. monocytogenes infected mice were stimulated with Yptb-βla for 2 hours and given antibiotics. Vγ1.1/2- CD44hi CD27- γδ T cells were analyzed for Yop translocation 2 hours post stimulation at the indicated MOI and quantified for Yop translocation. Data consists of one experiment with 2–10 mice/group and the graphs depict the mean ± SEM in (A-C). ****p < 0.0001, ***p < 0.001, and **p < 0.01. A t-test was used for (A), and a repeated measures one-way ANOVA was used for (C). Comparisons were performed to ΔYopB Y. pseudotuberculosis in (A) and as depicted in (C). (TIF) [file ppat.1010103.s002.tif]

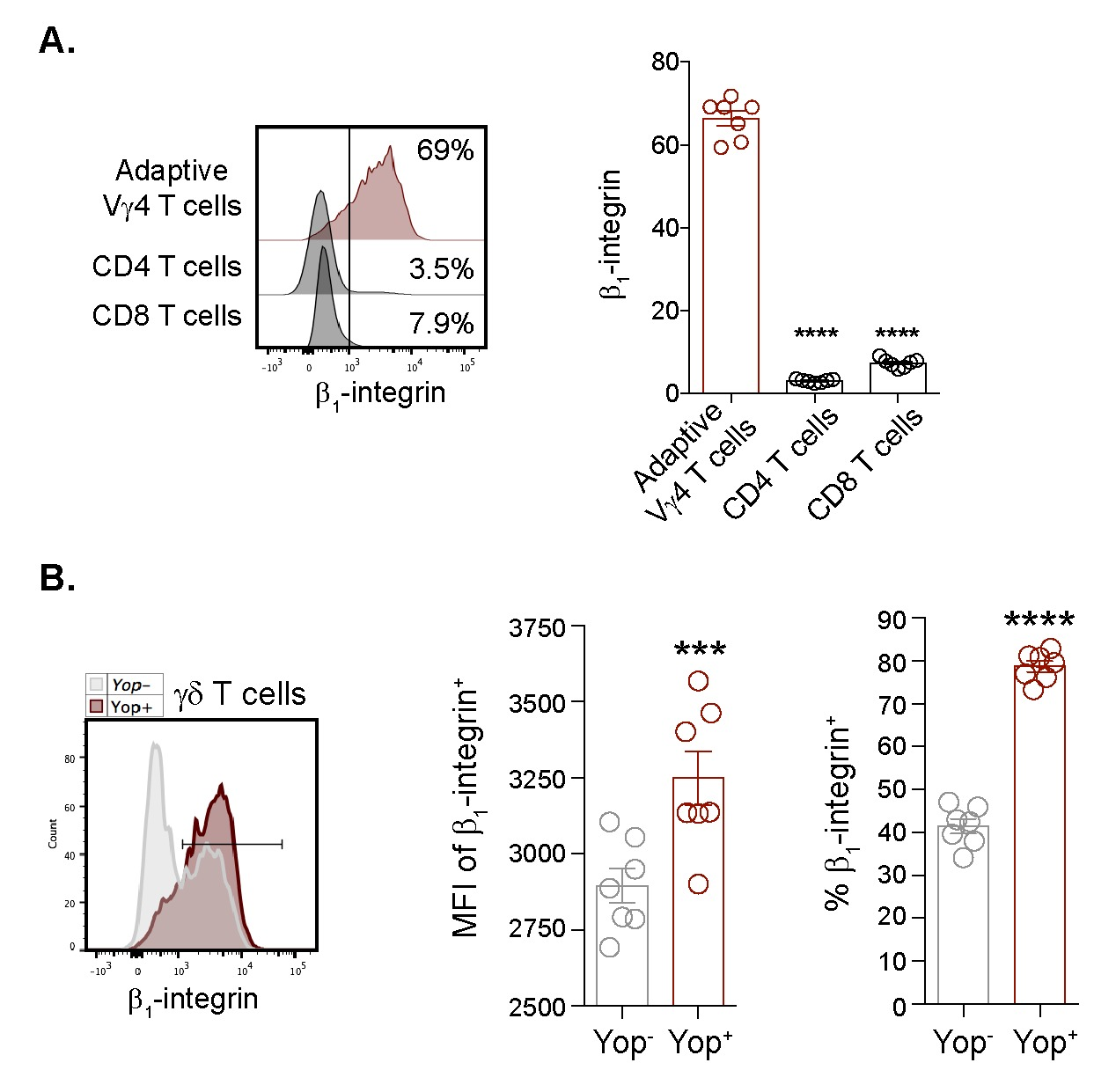

Supplement: S2 Fig — (A) MLN from L. monocytogenes infected mice were isolated and processed into single cell suspensions. Vγ1.1/2- CD44hi CD27- γδ T cells, CD4 T cells, and CD8 T cells were analyzed for β1-integrin expression. (B) MLN suspensions from L. monocytogenes infected mice were loaded with CCF4-AM dye and stimulated with 10 MOI WT Y. pseudotuberculosis containing a β-lactamase translocation reporter. CCF4-AM dye reports the occurrence of β-lactamase activity and Yop translocation. γδ T cells that contain Yop (Yop+) or do not contain Yop (Yop-) were analyzed for β1-integrin expression 2 hours after stimulation. Representative histogram plots are displayed. Data is pooled from two experiments with a total of 7 mice/group and the graphs depict the mean ± SEM in (A-C). ****p < 0.0001 and ***p < 0.001. A repeated measures one-way ANOVA was used for (A) and a t-test was used for (B), and. Comparisons were done to adaptive γδ T cells in (A) and as depicted in (B). (TIF) [file ppat.1010103.s003.tif]

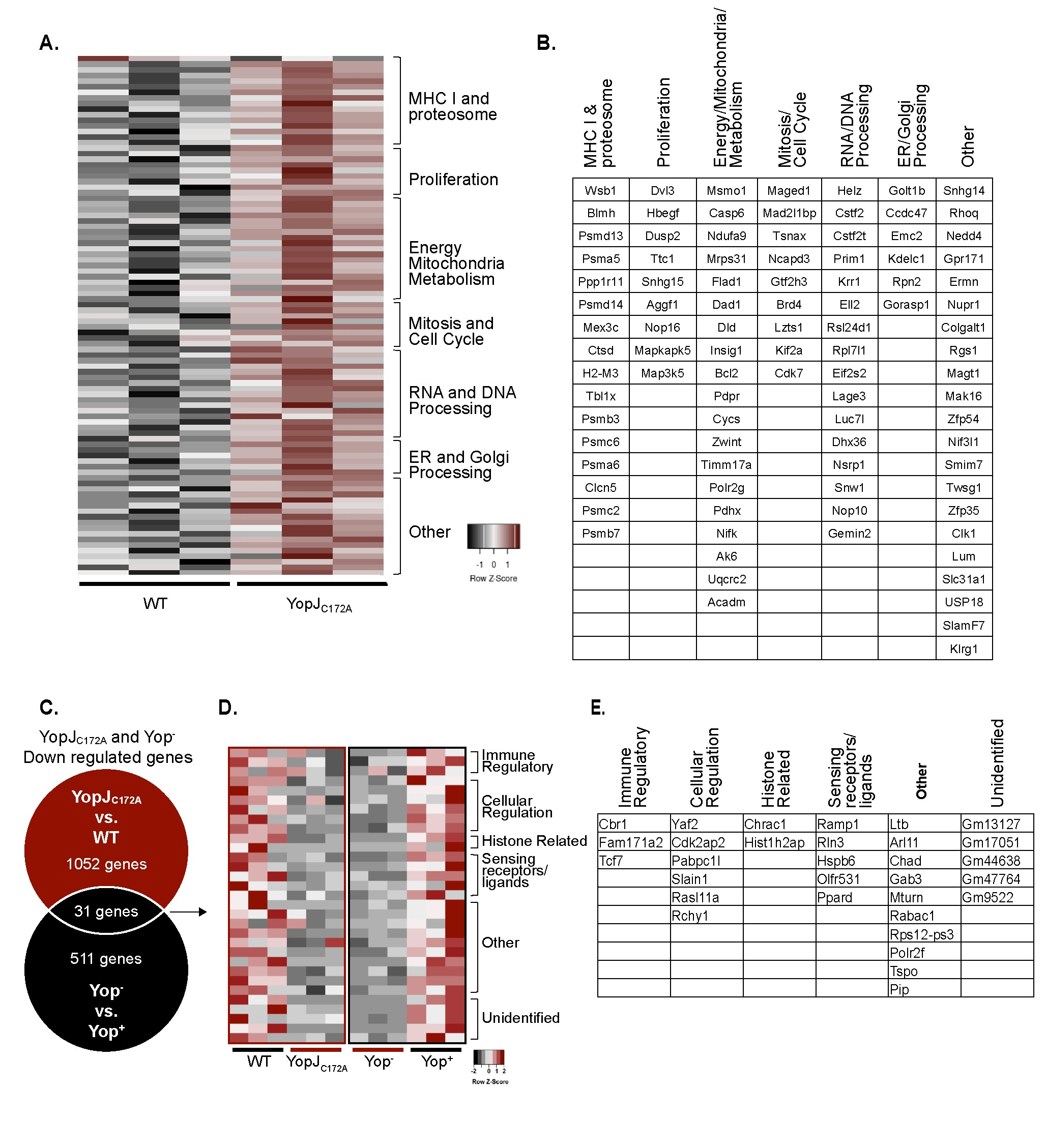

Supplement: S3 Fig — (A and B) MLN from L. monocytogenes infected mice were stimulated with 10 MOI of WT Y. pseudotuberculosis (WT) or mutant YopJ Y. pseudotuberculosis (YopJC172A) for 24 hours. Antibiotics were given 2 hours post-stimulation. Five hundred Vγ1.1/2- CD44hi CD27- γδ T cells from each stimulation were flow sorted and processed for RNA sequencing. The heat map depicts upregulated genes in Vγ1.1/2- CD44hi CD27- γδ T cells after YopJC172A Y. pseudotuberculosis stimulation and individual genes are listed. (C-E) Genes differentially expressed (downregulated) that overlapped between RNA sequencing analyses as displayed in the Venn diagram in (C) to select for direct effects of YopJ on Vγ1.1/2- CD44hi CD27- γδ T cells. The heat map depicts downregulated genes in Vγ1.1/2- CD44hi CD27- γδ T cells from the analysis in (C). Individual genes are listed in (E). Each experiment was performed once with biologic replicates. The cutoff for gene significance was p < 0.05 and FDR < 0.10. (TIF) [file ppat.1010103.s004.tif]

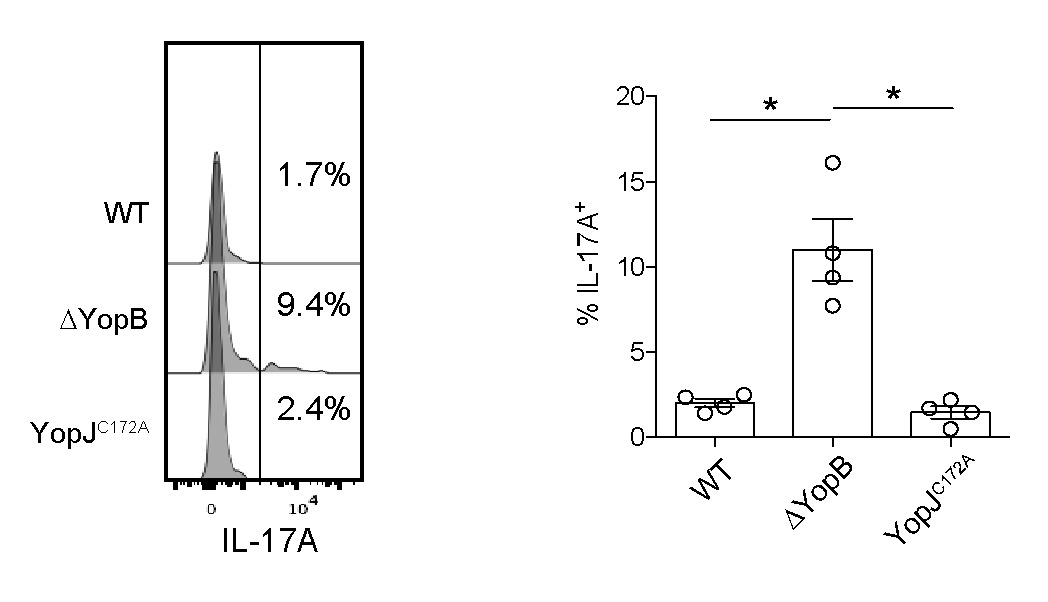

Supplement: S4 Fig — MLN cell suspensions from L. monocytogenes infected mice were stimulated with 10 MOI of WT, YopJC172A, or ΔYopB Y. pseudotuberculosis for 24 hours. Antibiotics were given 2 hours after stimulation and brefeldin A was added for the last 5–6 hours. Vγ1.1/2- CD44hi CD27- γδ T cells were analyzed for IL-17A production after stimulation. Representative histograms are displayed. The graph depicts mean ± SEM and represents two independent experiments with 4 mice per group. A repeated measures one-way ANOVA was used. * p < 0.05. (TIF) [file ppat.1010103.s005.tif]

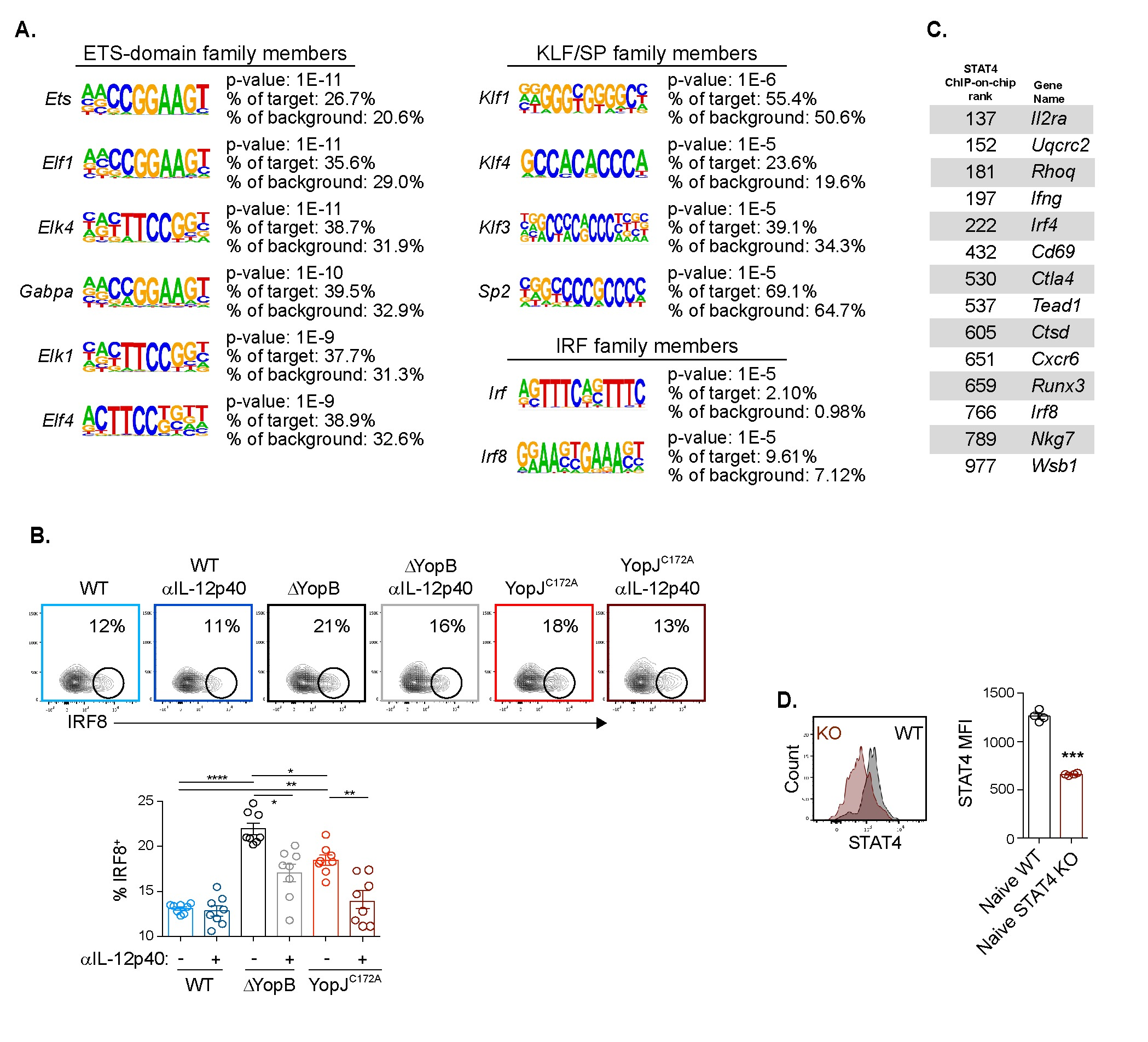

Supplement: S5 Fig — (A) Homer motif analysis was performed on the RNA sequencing results for the YopJC172A and WT Y. pseudotuberculosis comparison from Fig 5. The panel highlights the top transcription factor motifs of the ETS, SP/KLF, and IRF family of proteins identified in YopJC172A stimulated Vγ1.1/2- CD44hi CD27- γδ T cells. (B) MLN from L. monocytogenes infected mice were stimulated with 10 MOI of WT, YopJC172A, or ΔYopB Y. pseudotuberculosis for 6 hours. Antibiotics were given 2 hours post-stimulation. Vγ1.1/2- CD44hi CD27- γδ T cells were analyzed for IRF8 levels with or without anti-IL12p40 neutralizing antibody. The graph depicts the percentage of IRF8 protein expression among Vγ1.1/2- CD44hi CD27- γδ T cells after WT, YopJC172A, or ΔYopB Y. pseudotuberculosis stimulation. Data depict two pooled experiments with a total of 8 mice/group and represents the mean ± SEM. (C) The genes from the RNAseq and Homer motif analysis in Figs 4F and 4G and S3B and S5A were compared to an existing STAT4 ChIP-on-chip dataset to identify common genes. Genes from our dataset that were represented in the top 1000 genes of the Chip-on-chip dataset are displayed. (D) STAT4 KO spleens are shown in maroon and WT spleens are shown in black in representative histograms. The graph depicts the MFI of STAT4 protein expression in bulk γδ T cells. Data depicts one experiment with 4 mice/group and represents the mean ± SEM. ****p < 0.0001, ***p < 0.001, **p < 0.01, *p < 0.05. A repeated measures one-way ANOVA was used for (B), and a t-test was used for (D). Comparisons were performed as depicted in (B) and to Naïve WT in (D). (TIF) [file ppat.1010103.s006.tif]

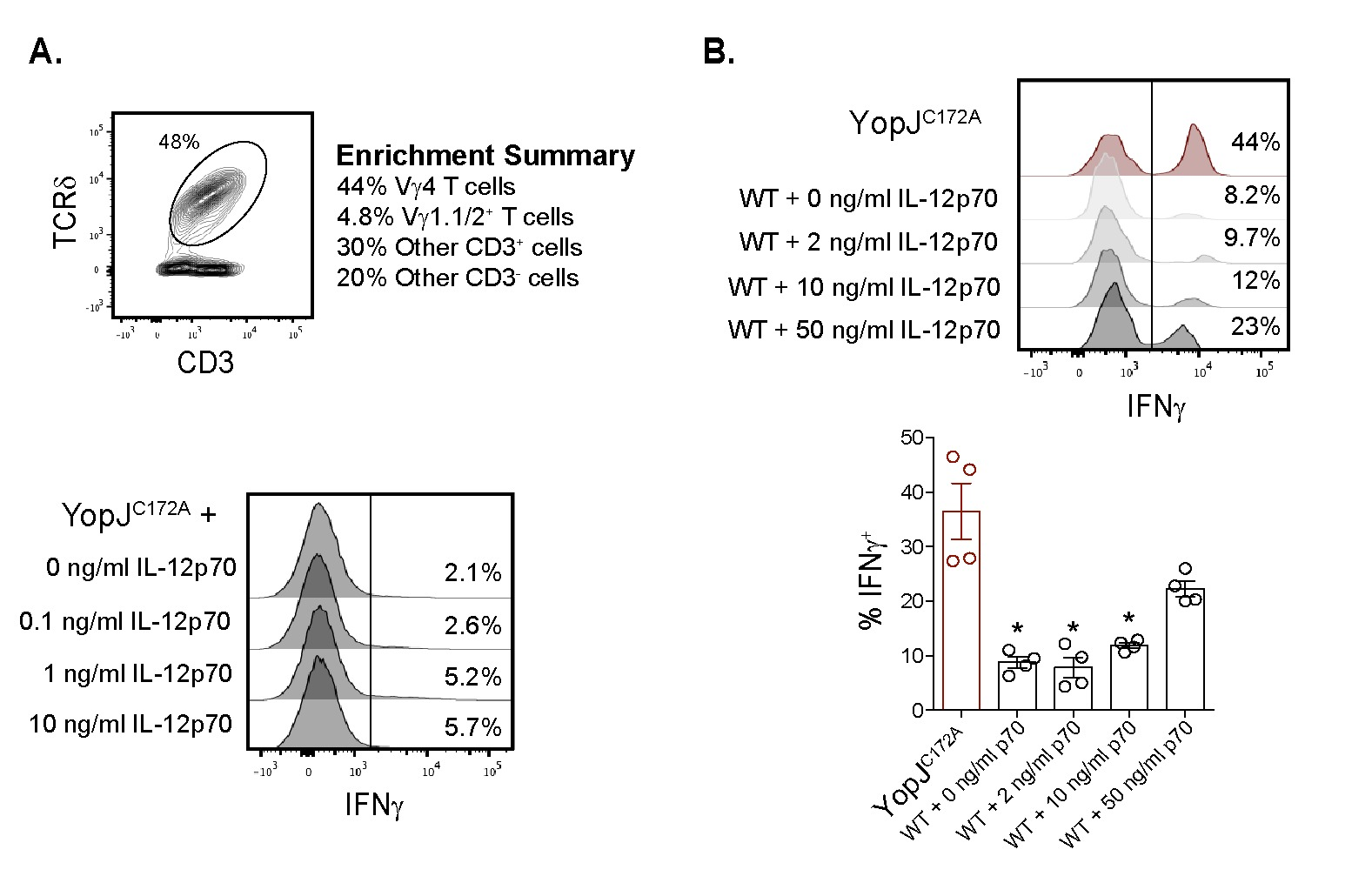

Supplement: S6 Fig — (A) γδ T cells enriched from the MLN and spleen of L. monocytogenes infected mice were expanded with plate bound γδTCR antibody for 2 days and rested for 5 days. After expansion, ~ 50% of cells were γδ T cells, and the majority of those were Vγ4 T cells. The enrichment summary reflects the mean enrichment from 4 samples. Afterwards, γδ T cells were isolated from cultures and stimulated with YopJC172A Y. pseudotuberculosis with 0.1, 1, or 10 ng/ml IL-12p70 for 24 hours. Antibiotics were added 2 hours after stimulation and brefeldin A was added for the last 5–6 hours. Histograms display IFNγ production from Vγ1.1/2- CD44hi CD27- γδ T cells under different culture conditions. Data depicts one experiment with 4 mice pooled and split into the indicated stimulation conditions. (B) MLN cell suspensions from L. monocytogenes infected mice were stimulated with 10 MOI of WT Y. pseudotuberculosis in the presence of 2, 10, or 50 ng/ml IL-12p70 or 10 MOI of YopJC172A Y. pseudotuberculosis for 24 hours. Antibiotics were given 2 hours after stimulation and brefeldin A was added for the last 5–6 hours. Vγ1.1/2- CD44hi CD27- γδ T cells were analyzed for IFNγ production. Representative histograms of IFNγ production from Vγ1.1/2- CD44hi CD27- γδ T cells are displayed. The graph depicts mean ± SEM from one experiment with 4 mice per group *p < 0.05. A repeated measures one-way ANOVA was used for comparisons to YopJC172A Y. pseudotuberculosis in (B). (TIF) [file ppat.1010103.s007.tif]

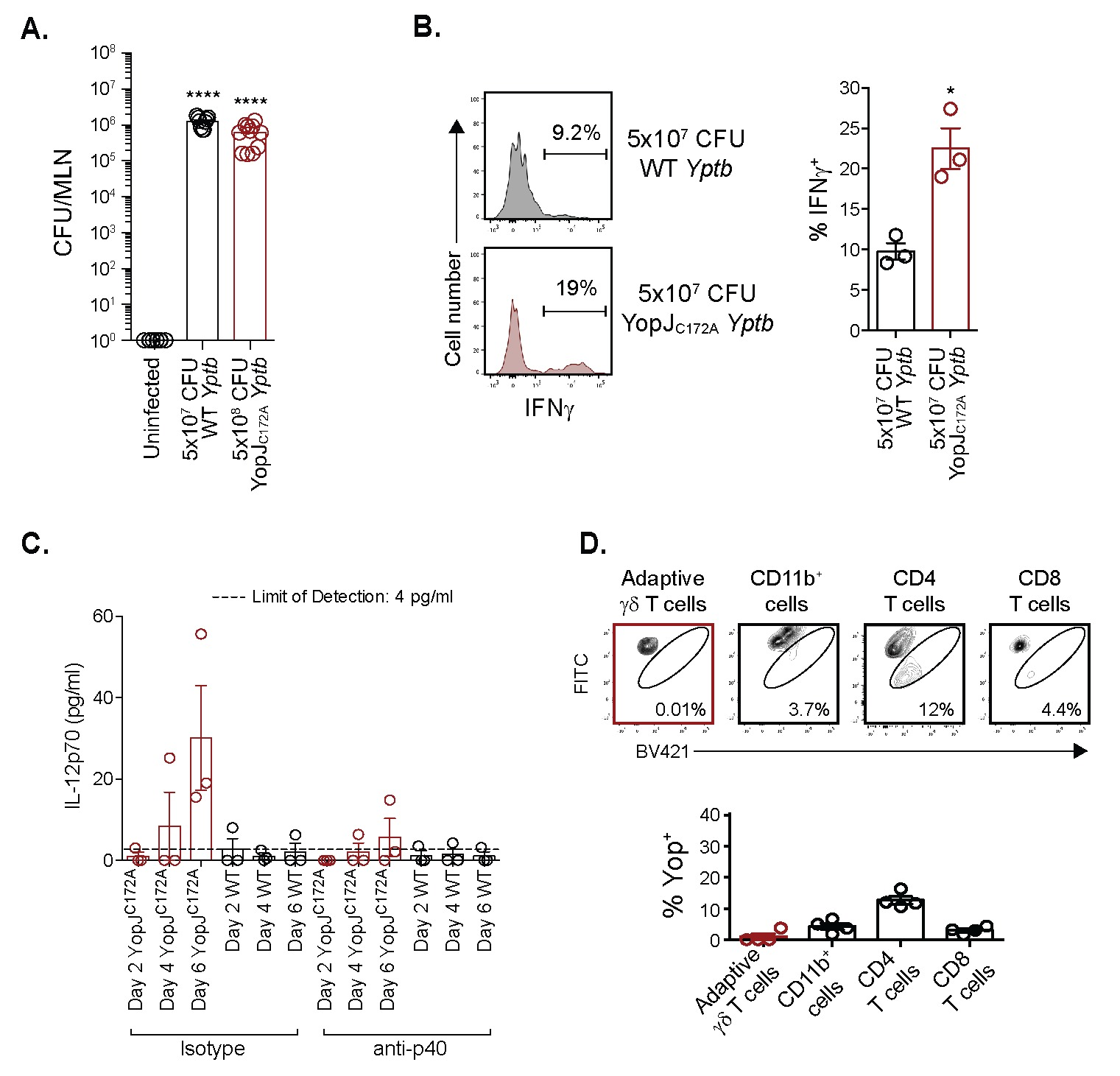

Supplement: S7 Fig — (A) Balb/c mice were foodborne infected with the indicated doses of WT or mutant YopJC172A Y. pseudotuberculosis and tissues were analyzed 9 days post-infection. Bacteria burden was quantified from the MLN. Data reflect 3–5 mice per group pooled from 3 independent experiments and the graphs depict the mean ± SEM. (B) Balb/c mice were foodborne infected with the indicated doses of WT or mutant YopJC172A Y. pseudotuberculosis. Nine days post infection, MLN suspensions from WT or YopJC172A Y. pseudotuberculosis infected mice were stimulated with PMA/ionomycin and brefeldin A for 4 hours. Vγ1.1/2- CD44hi CD27- γδ T cells were analyzed for IFNγ production. Representative histograms are displayed and quantified. Data depicts one experiment with 3 mice per group. (C) Balb/c mice were foodborne infected with WT (2-4x10^7^ CFU) or YopJC172A Y. pseudotuberculosis (2-4x10^8^ CFU) and treated with 0.2 mg/mouse of anti-IL12p40 on days 0, 2, 4, and 6 post infection. IL-12p70 concentrations were determined from serum at days 2, 4, and 6 post infection. Data represent 2 independent experiments with a total of 9 mice per group. Serum samples were pooled into groups of 3 per experimental condition. (D) Balb/c mice were foodborne infected with 2x10^9^ CFU L. monocytogenes to elicit a Vγ1.1/2- CD44hi CD27- γδ T cell population in vivo. 30 days post infection, adaptive Vγ1.1/2- CD44hi CD27- γδ T cells from the MLN of immune mice were analyzed for Yop translocation using the CCF4-AM assay. Representative contour plots are shown. Yop translocation (Yop+) among the indicated populations represents background staining as a negative control for Fig 6F. The graph depicts the mean ± SEM and is pooled from 2 experiments with a total of 4 mice per group. ****p < 0.0001, *p < 0.05. A one-way ANOVA was used for (A), and an unpaired t-test was used for (B). Comparisons were performed to uninfected in (A) and to 5x10^7^ WT Y. pseudotuberculosis in (B). (TIF) [file ppat.1010103.s008.tif]
